# Distinct origins and niches determine the cellular responsiveness of CNS macrophages after repopulation

**DOI:** 10.1038/s41590-026-02457-y

**Published:** 2026-03-18

**Authors:** Maximilian Fliegauf, Damien Levard, Francesco Cardamone, Maximilian Frosch, Siegmar Kuhn, Roman Sankowski, Martino Provinciali, Anaelle Aurelie Dumas, Adrià Dalmau Gasull, Philipp Aktories, Alexander Oschwald, Marius Schwabenland, Rebekka Scholz, Marc D. Beyer, Jonas J. Neher, Michael Kollefrath, Dimos Baltas, Wilfried Reichardt, Dominik von Elverfeldt, Denis Vivien, Jovana Cupovic, Nicola Iovino, Marina Rubio, Lukas Amann, Marco Prinz

**Affiliations:** 1 Institute of Neuropathology, Medical Center and Faculty of Medicine, University of Freiburg, Freiburg, Germany; 2 Department of Pharmaceutical Biology and Biotechnology, Institute of Pharmaceutical Sciences, University of Freiburg, Freiburg, Germany; 3 Normandie University, UNICAEN, Université Caen Normandie, INSERM UMR-S U1237, Physiopathology and Imaging of Neurological Disorders (PhIND), GIP Cyceron, Institut Blood and Brain @ Caen-Normandie (BB@C), Caen, France; 4 Max Planck Institute of Immunobiology and Epigenetics, Freiburg, Germany; 5 Faculty of Biology, University of Freiburg, Freiburg, Germany; 6 International Max Planck Research School of Immunobiology, Epigenetics and Metabolism (IMPRS-IEM), Freiburg, Germany; 7 Division of Medical Physics, Department of Radiation Oncology, Medical Center and Faculty of Medicine, University of Freiburg, Freiburg, Germany; 8 Berta-Ottenstein-Programme for Clinician Scientists, Faculty of Medicine, University of Freiburg, Freiburg, Germany; 9 Immunogenomics & Neurodegeneration, German Center for Neurodegenerative Diseases (DZNE), Bonn, Germany; 10 Systems Medicine, German Center for Neurodegenerative Diseases (DZNE), Bonn, Germany; 11 PRECISE Platform for Single Cell Genomics and Epigenomics, DZNE and University of Bonn and West German Genome Center, Bonn, Germany; 12 German Center for Neurodegenerative Diseases, Munich, Germany; 13 Biomedical Center (BMC), Biochemistry, Faculty of Medicine, LMU Munich, Munich, Germany; 14 Munich Cluster for Systems Neurology (SyNergy), Munich, Germany; 15 German Cancer Consortium (DKTK), Partner Site DKTK, Freiburg, Germany; 16 Division of Medical Physics, Department of Diagnostic and Interventional Radiology, University Medical Center Freiburg, Faculty of Medicine, University of Freiburg, Freiburg, Germany; 17 Department of Clinical Research, Caen-Normandie University Hospital, CHU, Caen, France; 18 Signalling Research Centres BIOSS and CIBSS, University of Freiburg, Freiburg, Germany

**Keywords:** Neuroimmunology, Innate immune cells

## Abstract

Nonparenchymal central nervous system (CNS)-associated macrophages (CAMs) mediate immune responses at brain boundaries. Perivascular and leptomeningeal CAMs are collectively termed subdural CAMs (sdCAMs). Both sdCAMs and juxtaneuronal microglia are derived from embryonic yolk sac precursors, long-living and maintain their populations through self-renewal. Following depletion, microglia autonomously repopulate from single surviving cells. In contrast, the course of sdCAM repopulation remains poorly understood. Here, by combining multilineage fate mapping, multiomic profiling and high-resolution imaging, we demonstrate divergent repopulation dynamics between sdCAMs and microglia. Unlike microglia, sdCAMs do not renew cell-autonomously, but become transiently accessible to CCR2^+^Ly6C^+^ monocyte engraftment after niche induction in an integrin-dependent manner. Moreover, replenished monocyte-derived sdCAMs remain transcriptomically, epigenetically and functionally distinct from their embryo-derived counterparts. Finally, we present a protocol enabling selective exchange of sdCAMs, modulating disease response without functionally affecting microglia. These new insights into CNS immune biology suggest new therapeutic avenues for neuroinflammatory and neurodegenerative diseases.

## Abstract (web summary)

Prinz and colleagues examine the distinct functions and repopulation properties of leptomeningeal and perivascular macrophages that mediate immune responses at brain boundaries.

## Main

The central nervous system (CNS) hosts different tissue-resident macrophages. Microglia seed the parenchyma, whereas CNS-associated macrophages (CAMs)^1^, also known as border-associated macrophages (BAMs)^2^, are found at the CNS interfaces. CAMs have been further subdivided based on their anatomical location. The outermost meningeal layer of the CNS, the dura mater, is home to dural CAMs (dCAMs)^3^. Subdural CAMs (sdCAMs) include perivascular macrophages (pvMΦ), positioned in the Virchow-Robin space, as well as leptomeningeal macrophages (lmMΦ) and are thus situated at the parenchymal surface^4^, hence their alternative naming as parenchymal border macrophages^5^. CNS macrophages are involved in a plethora of perturbations such as inflammatory, degenerative or neurooncological disorders, where they shape the disease course^1,6–8^.

Microglia and lmMΦ originate during embryogenesis from a common c-Kit^+^ erythromyeloid progenitor in the extraembryonic YS^4,9–11^. PvMΦ are part of the lmMΦ pedigree, as they emerge during the early postnatal phase from descending lmMΦ that migrate into the developing Virchow-Robin space in a talin-1-mediated fashion^12^. Of note, and in contrast to other CAM subsets such as dCAMs, microglia, lmMΦ and pvMΦ are self-maintaining throughout life under homeostatic conditions and are not replaced by any circulating blood cells^3,4,13–15^.

For their survival, microglia and CAMs depend on constant signaling through the colony-stimulating factor 1 receptor (CSF-1R). Consequently, genetic deletion as well as blockade of CSF-1R signaling by inhibitors, such as PLX3397, PLX5622 or BLZ945, leads to effective depletion of both microglia and CAMs^9,16–19^. Previous studies employing various depletion strategies elucidated the repopulation features of microglia in mice^20–22^. Through fate mapping-based lineage-tracing, these studies demonstrated that, following a single depletion course, repopulated microglia emerge exclusively from the proliferation of surviving microglia without input from circulating blood cells^18,22^. Only if the endogenous microglial compartment is continuously or severely compromised, can monocytes replace microglia, representing an exception to the physiological rule^23,24^.

While depletion and repopulation of microglia is being actively tested as a therapeutic strategy for microgliopathies^25–28^, similar studies for CAMs are still very sparse^18,29,30^. Their low abundance, combined with the historical lack of tools to comprehensively and selectively investigate them, has hindered the study of the cellular sources, underlying molecular mechanisms and disease-related functional consequences of CAM repopulation.

## Divergent spatiotemporal replenishment of CAMs and microglia

**Fig. 1:**
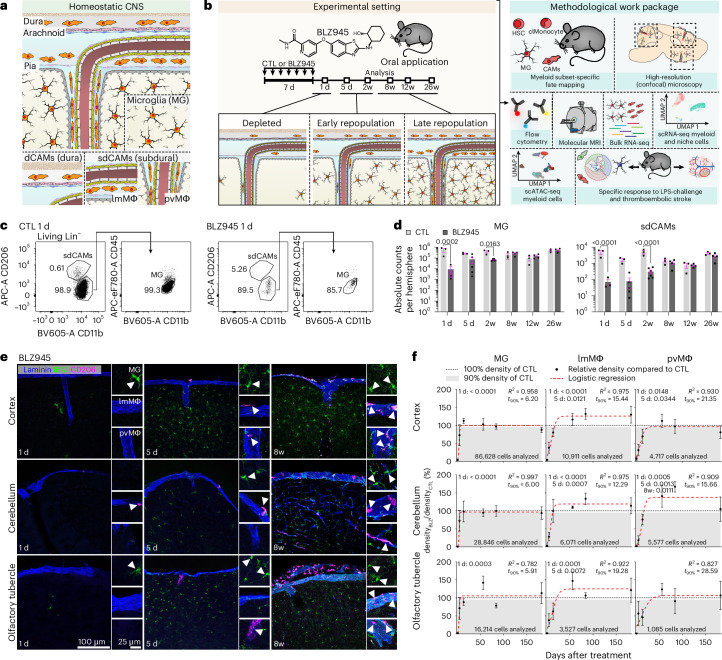
Spatially and temporally distinct repopulation kinetics of CAMs and MG. **a**, Overview of microglia (MG) and subsets of CAMs during homeostasis in the CNS. dCAMs, dural CAMs; sdCAMs, subdural CAMs, including leptomeningeal (lmMΦ) and perivascular (pvMΦ) macrophages. **b**, Left: Scheme of the experimental layout. Right: CNS macrophage repopulation in complementary myeloid-specific fate mapping mouse lines was analyzed by confocal microscopy, flow cytometry, RNA-seq and scATAC-seq, followed by systemic LPS challenge and a middle-cerebral artery occlusion (MCAO) model of thromboembolic stroke. **c**,**d**, Representative flow cytometry plots of MG and sdCAMs after depletion (**c**) and quantification of their numbers per brain hemisphere (**d**). Symbols represent individual mice, *n* = 3 (CTL and 1 d BLZ945), *n* = 4 (8w, 12w, 26w BLZ945), *n* = 5 (5 d BLZ945), *n* = 6 (2w BLZ945), mean ± s.e.m. Ordinary one-way analysis of variance (ANOVA) with Šidák-adjusted multiple-comparisons *P* values. **e**, Representative confocal images from cerebral cortex, cerebellum and olfactory tubercle at 1 d, 5 d and 8w after BLZ945 treatment in WT mice. Arrows point to myeloid cells in their distinct compartments. **f**, Proportion of MG, lmMΦ and pvMΦ density at different time points and in diverse brain regions after BLZ945 treatment compared to controls (CTL). Symbols represent mean depletion at each time point, calculated as proportion of CTL mean. Error bars indicate s.e. of the depletion estimate, propagated from the standard errors of BLZ945 and CTL densities. *n* = 3 (CTL and 1 d BLZ945), *n* = 4 (8w, 12w, 26w BLZ945), *n* = 5 (5 d BLZ945), *n* = 6 (2w BLZ945), logistic growth modeling, *t*_90%_: time point in days when the repopulation reaches 90% compared to controls. *R*^2^: coefficient of determination for model equation and data points). Unpaired two-tailed Welch’s *t*-test with Bonferroni-corrected *P* values. Source data

Following a single transient depletion, microglia rapidly repopulate, indicating a high demand for replenishing the juxtaneuronal niche^20,21^. To investigate the renewal kinetics of CAMs (Fig. 1a), we temporarily administered BLZ945, a brain penetrant CSF-1R inhibitor, to wild-type (WT) mice (Fig. 1b)^16,17^.

Flow cytometry-based cell quantification of the whole brain revealed a robust reduction of microglia and sdCAM numbers compared to controls (Fig. 1c, d). While microglia had mostly recovered after 5 days (5 d) of repopulation, sdCAMs were still largely absent. At 8 weeks (8w) after depletion; however, both microglia and sdCAM numbers were fully restored. As microglial self-renewal kinetics differ between brain regions^13,31^, we examined the repopulation of IBA1^+^CD206^−^ microglia as well as CD206^+^ lmMΦ and pvMΦ in cortex, cerebellum and olfactory tubercle (Fig. 1e, f). Histological assessment confirmed successful depletion of both microglia and sdCAMs at 1 d, followed by a swift re-emergence of microglia, whereas only few lmMΦ and pvMΦ were observed early during repopulation (Fig. 1e, f). An evaluation of the renewal kinetics revealed quick and uniform re-establishment of microglia (*t*_90%_ of controls: 6.20 d in cerebral cortex, 6.00 d in cerebellum, 5.91 d in olfactory tubercle). In sharp contrast, repopulation of both lmMΦ and pvMΦ occurred at greatly reduced velocity and in a spatially distinct manner (*t*_90%_ for lmMΦ, 15.44 d cerebral cortex, 12.29 d cerebellum, 19.28 d olfactory tubercle; and *t*_90%_ for pvMΦ, 21.35 d, 15.66 d, 28.59 d, respectively).

## Repopulating sdCAMs intensify cell–cell communications

**Fig. 2:**
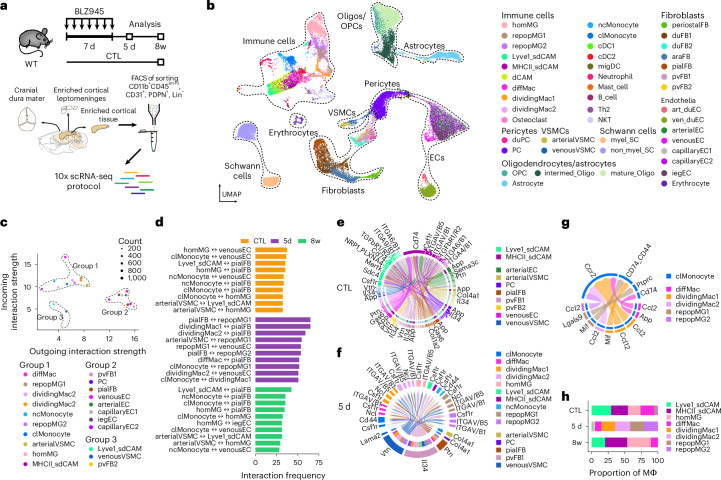
Repopulating CNS macrophages upregulate chemokines while their niche remains largely unchanged. **a**, Experimental scheme: C57BL/6 mice were treated either with BLZ945 or kept as controls (CTL). Cranial dura mater, enriched cortical leptomeninges and cortex without meninges were isolated and CD11b^+^CD45^int-hi^, PDPN^+^, CD31^+^ and Lin^−^ cells were sorted for scRNA-seq. *n* = 4 (dura 8w and 5 d), *n* = 3 (dura CTL), *n* = 8 (leptomeninges and cortex 8w and 5 d), *n* = 7 (leptomeninges and cortex CTL). **b**, Uniform Manifold Approximation and Projection (UMAP) of 39,838 individual cells with cell type annotation. **c**, Predicted incoming and outgoing interaction strengths for all cell types from leptomeninges and cortex were calculated using CellChat. Size of dots represents number of predicted interactions. **d**, Top ten predicted interaction partners by frequency of interaction between myeloid cell types and their surrounding niche in leptomeninges and cortex by time point (CTL, 5 d, 8w). **e**, Chord plot depicting predicted interactions between the two homeostatic CAM subtypes and the top interacting niche cell types. **f**,**g**, Chord plot depicting interactions between macrophages and either niche cells (**f**) or clMonocytes (**g**) that are differentially upregulated at 5 d after BLZ945. **h**, Proportion of macrophage clusters among all macrophages in leptomeninges and cortex samples. Proportions are derived from averages across leptomeningeal and cortical samples. ara, arachnoid; art, arterial; diff, differentiating; du, dural; EC, endothelial cells; FB, fibroblast; hom, homeostatic; ieg, immediate early genes; Mac, macrophage; myel, myelinating; nc, non-classical; Oligo, oligodendrocytes; PC, pericytes; repop, repopulating; SC, Schwann cells; ven, venous; VSMC, vascular smooth muscle cell; OPC, oligodendrocyte progenitor cell; cDC1/2, classical dendritic cell 1/2; migDC, migratory dendritic cell; NKT, natural killer T cell; Th2, T helper 2 cell.

**Extended Data Fig. 1:**
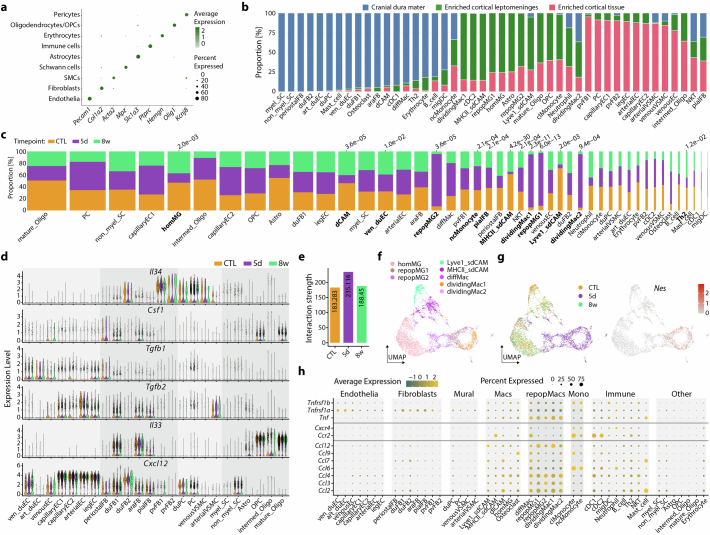
Single-cell RNA-sequencing of the CNS macrophage niche. (**a**) Dot plot depicting canonical cell-type-defining markers. Color scale: Relative expression level. (**b**) Bar graph showing the proportion of each cluster within the different tissues (Related to Fig. 2). (**c**) Marimekko chart showing the proportion of each cluster within each treatment group. CTL: control; 5d: five days after BLZ945; 8w: eight weeks after BLZ945. *n* = 4 (Dura 8w and 5d), *n* = 3 (Dura CTL), *n* = 8 (Leptomeninges and Cortex 8w and 5 d), *n* = 7 (Leptomeninges and Cortex CTL), arcsine transformation followed by ordinary one-way ANOVA combined with Benjamini–Hochberg FDR correction. (**d**) Violin plots showing expression levels of selected macrophage-niche factors and chemokines across niche cells. (**e**) Bar plot showing aggregated strength of all interactions at the different analysis time points. (**f, g**) Reclustered UMAP of all macrophage subsets in leptomeninges and cortex (**f**). Feature plots of condition (**g**, left) and *Nes* (*Nestin*) expression levels (**g**, right) are shown. Scale bar shows z-score of minimum to maximum scaled gene expression. (**h**) Dot plot depicting gene expression of a selection of tumor necrosis factor (TNF) signaling components and chemokines predicted to mediate interactions between myeloid and niche cells. Color scale: Relative expression levels. Source data

CNS macrophages closely interact with diverse neighboring cells, forming a niche that provides essential signals shaping their phenotype, function, and survival^1,32^. To investigate the local tissue environments during repopulation, we performed single-cell RNA-sequencing (scRNA-seq) of CD11b^+^CD45^int-hi^ myeloid cells together with CD31^+^ endothelial cells, nonhematopoietic PDPN^+^ as well as lineage negative (Lin^−^) cells from micro-dissected CNS compartments of control mice after depletion (Fig. 2a and Supplementary Fig. 1a). Analyzing 39,838 individual cells (Fig. 2b), we classified immune and niche cells according to canonical marker expression (Extended Data Fig. 1a), their tissue of origin (Extended Data Fig. 1b) and cell subtype markers previously identified^3,33–37^ (Supplementary Fig. 1b). We found that depletion and repopulation significantly affected macrophage abundance and composition with little impact on niche cells or other immune cells (Extended Data Fig. 1c). At 5 d after depletion, both homeostatic microglia and CAM populations were reduced, whereas repopulating macrophage subtypes (dividing Mac1, dividing Mac2, repop MG1 and repop MG2) arose, characterized by expression of *Mki67* and other cell-cycle genes (Supplementary Fig. 1b). Differential expression analysis revealed only mild effects of CNS macrophage depletion and repopulation on gene expression in niche cells (Supplementary Fig. 2). Similarly, expression levels of factors known to regulate CNS macrophage survival, phenotype and function showed no clear differences (Extended Data Fig. 1d).

To further explore local macrophage-niche interactions dynamics in the subdural compartment, we deployed CellChat. Assessing predicted outgoing versus incoming signaling strength, we visually identified three clusters of cells (Fig. 2c). Group 1, mainly composed of macrophages, were predicted to be the strongest signal receivers, with repopulating subtypes showing higher outgoing interaction strength than homeostatic counterparts. Group 2, including fibroblasts, endothelial cells and pericytes, were predicted to act as strong senders, whereas group 3 showed low interaction strength (Fig. 2c). The overall interaction strength showed a spike at 5 d post-depletion and a return to baseline 8w after depletion (Extended Data Fig. 1e). When investigating the contribution of niche-macrophage–monocyte interactions to this phenomenon in the subdural compartment, we found the highest interaction frequencies between repopulating macrophage subtypes and the vascular and leptomeningeal stroma (Fig. 2d). In control animals, signaling pathways from endothelial, mural and fibroblastic stromal cells contributed in a balanced way to sdCAM niche homeostasis (Fig. 2e). Assessing pathways upregulated during repopulation, we identified perivascular fibroblast-derived *Il34* as the most prominent signal received by repopulating sdCAMs (Fig. 2f).

By examination of subclustered subdural macrophage populations we found early repopulating CNS macrophages to express *Nestin*, in agreement with previous findings^22^ (Extended Data Fig. 1f, g). Repopulating subdural macrophage subsets and classical monocytes (clMonocyte) were among the cell types with highest interaction frequencies at 5 d (Fig. 2d). Between these cells, we found CCR2-mediated signaling, a canonical monocyte attraction pathway, to be the most prominent predicted interaction (Fig. 2g). Furthermore, repopulating CNS macrophages highly expressed *Tnf* and chemokines, including *Ccl3*, *Ccl4*, *Ccl6*, *Ccl7* and *Ccl9*, whereas the corresponding receptors were found on monocytes and endothelial cells (Extended Data Fig. 1h). Notably, the relative abundance of sdCAM subtypes had changed 8w after depletion compared to controls (Fig. 2h). Taken together, transcriptional profiling revealed that CNS macrophage-niche cells were largely unaffected; however, the change in sdCAM composition, combined with the upregulation of monocyte-attracting chemokines, led us to investigate the cellular origins of sdCAM repopulation.

## sdCAMs and microglia renew from distinct cellular sources

**Extended Data Fig. 2:**
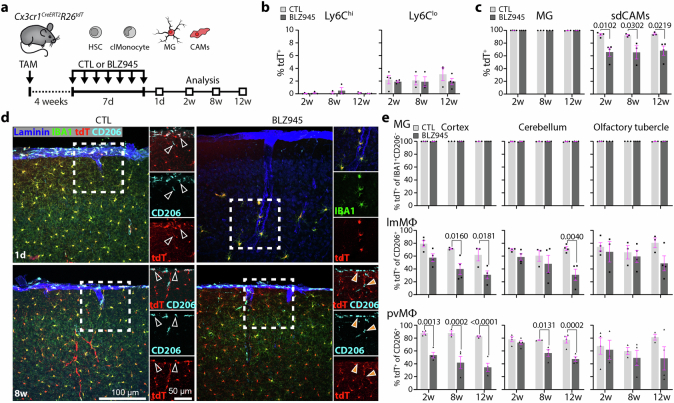
Non-cell-autonomous repopulation of sdCAMs but not MG throughout the brain. (**a**) Experimental scheme: Tamoxifen (TAM)-induced *Cx3cr1*^*CreERT2*^*R26*^*tdT*^ mice were treated with control (CTL) or BLZ945 and analyzed at different time points thereafter. (**b**) TdTomato (tdT) expression in Ly6C^hi^ and Ly6C^lo^ blood monocytes from *Cx3cr1*^*CreERT2*^*R26*^*tdT*^ mice. Symbols represent individual mice, *n* = 3 (8w, 12w CTL), *n* = 4 (2w, 12w BLZ945), mean ± SEM. Ordinary one-way ANOVA with Šidák-adjusted multiple-comparisons p values. (**c**) Flow cytometry-based quantification of labeling in microglia (MG) or sdCAMs. Symbols represent individual mice, *n* = 3 (8w, 12w CTL), *n* = 4 (2w, 12w BLZ945), mean ± SEM. Ordinary one-way ANOVA with Šidák-adjusted multiple-comparisons p values. (**d, e**) Representative images from cerebral cortex, cerebellum and olfactory tubercle at 1d and 8w post CTL or BLZ945 treatment, respectively. Arrowheads: blank = tdT^+^ lmMΦ or pvMΦ; orange = tdT^−^ lmMΦ or pvMΦ (**d**). Histological quantification of the proportion of tdT^+^ MG, lmMΦ and pvMΦ at different time points and in different brain regions (**e**). Symbols represent individual mice, *n* = 3 (8w, 12w CTL), *n* = 4 (2w, 12w BLZ945), mean ± SEM. Ordinary one-way ANOVA with Šidák-adjusted multiple-comparisons p values. Source data

To determine whether sdCAMs repopulate completely cell-autonomously, similarly to microglia^18,22^, we employed several myeloid fate mapping mouse models. First, we performed depletion experiments in *Cx3cr1*^*CreERT2*^*R26*^*tdT*^ mice (Extended Data Fig. 2a). As expected^4,33,38^, short living CX_3_CR1^+^Ly6C^hi^ monocytes quickly lost labeling after tamoxifen (TAM) injection (Extended Data Fig. 2b). In contrast, flow cytometry-based (Extended Data Fig. 2c) and histological analyses (Extended Data Fig. 2d, e) revealed constant high labeling in fully renewed microglia at all time points after depletion (Extended Data Fig. 2c). Conversely, sdCAMs showed a clear reduction in tdTomato^+^ (tdT^+^) proportion, with many sdCAMs remaining tdT^−^ 12w after depletion, suggesting noncell-autonomous sources of their repopulation.

**Fig. 3:**
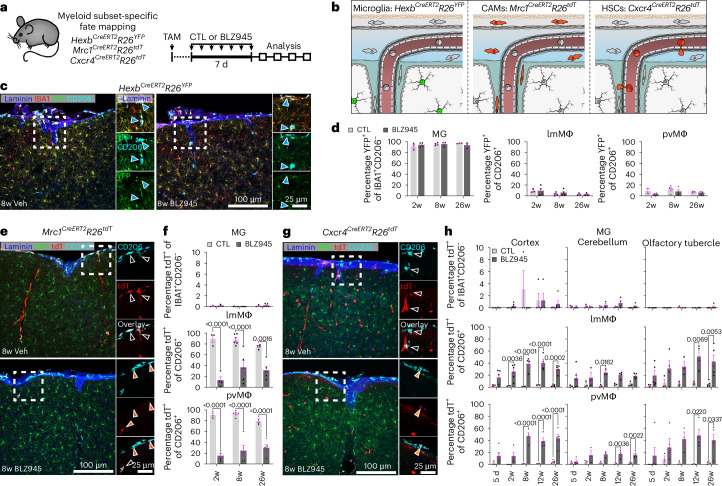
Compartment-specific contributions of hematopoiesis to CNS macrophage repopulation. **a**,**b**, Experimental scheme. **c**, Representative images of YFP labeling in cerebral cortex from *Hexb*^*CreERT2*^*R26*^*YFP*^ mice at 8w after CTL or BLZ945 treatment. Arrowheads depict CD206^+^YFP^−^ cells. **d**, Quantification of YFP labeled MG, lmMΦ and pvMΦ in *Hexb*^*CreERT2*^*R26*^*YFP*^ mice at different time points post CTL or BLZ945 treatment. Symbols represent individual mice, *n* = 2 (2w CTL pvMΦ), *n* = 3 (2w CTL MG and lmMΦ, 26w), *n* = 4 (2w BLZ945, 8w), mean ± s.e.m. Ordinary one-way ANOVA with Šidák-adjusted multiple-comparisons *P* values. **e**, Typical immunofluorescence images of tdT labeling in cerebral cortex from *Mrc1*^*CreERT2*^*R26*^*tdT*^ mice at 8w after treatment. Arrowheads, blank indicate CD206^+^tdT^+^ cells; orange show CD206^+^tdT^−^ cells. **f**, Quantification of tdT labeled MG, lmMΦ and pvMΦ in *Mrc1*^*CreERT2*^*R26*^*tdT*^ mice at different time points after treatment. Symbols represent individual mice, *n* = 3 (2w CTL), *n* = 4 (2w BLZ945 and 26w CTL), *n* = 5 (8w BLZ945 and 26w BLZ945) *n* = 6 (8w CTL), mean ± s.e.m. Ordinary one-way ANOVA with Šidák-adjusted multiple-comparisons *P* values. **g**, Representative pictures of tdT labeling in the cerebral cortex from *Cxcr4*^*CreERT2*^*R26*^*tdT*^ mice at 8w after treatment. Arrowheads, blank show CD206^+^tdT^−^ cells; orange show CD206^+^tdT^+^ cells. **h**, Quantification of tdT labeled proportions of MG, lmMΦ and pvMΦ in *Cxcr4*^*CreERT2*^*R26*^*tdT*^ mice at different time points after treatment in cerebral cortex, cerebellum and olfactory tubercle. Symbols represent individual mice, *n* = 3 (5 d CTL, 2w CTL, 8w CTL and 12w CTL), *n* = 4 (2w BLZ945, 8w BLZ945 and 12w BLZ945), *n* = 5 (5 d BLZ945, 26w), mean ± s.e.m. Ordinary one-way ANOVA with Šidák-adjusted multiple-comparisons *P* values. Source data

**Extended Data Fig. 3:**
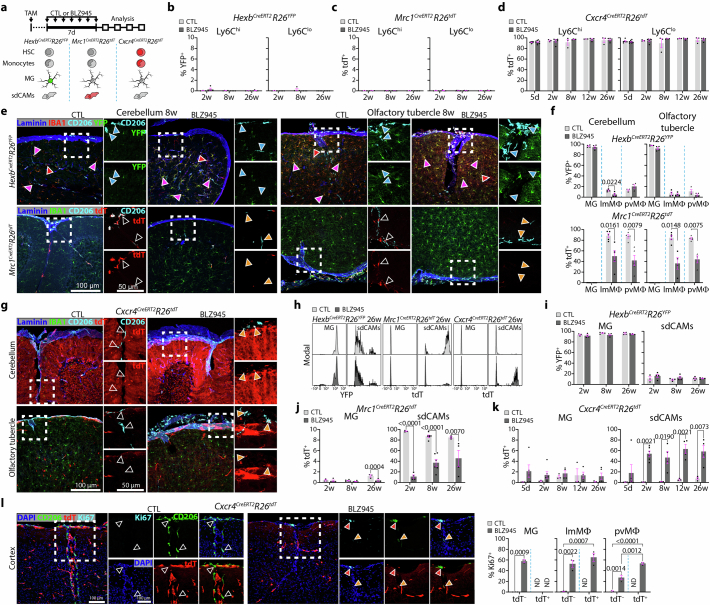
Repopulation dynamics of CNS macrophages throughout the brain. (**a**) Experimental scheme. HSC: hematopoietic stem cell; MG: microglia, Mono: monocyte, TAM: Tamoxifen. (**b-d**) Flow cytometric quantification of reporter gene expression in Ly6C^hi^ and Ly6C^lo^ blood monocytes from *Hexb*^*CreERT2*^*R26*^*YFP*^ (**b**), *Mrc1*^*CreERT2*^*R26*^*tdT*^ (**c**) and *Cxcr4*^*CreERT2*^*R26*^*tdT*^ (**d**). Symbols represent individual mice, *Hexb*^*CreERT2*^*R26*^*YFP*^: *n* = 3 (2w CTL), *n* = 4 (2w BLZ945, 8w, 26w), *Mrc1*^*CreERT2*^*R26*^*tdT*^: *n* = 3 (2w CTL, 26w CTL), *n* = 4 (2w BLZ945, 26w BLZ945), *n* = 6 (8w), *Cxcr4*^*CreERT2*^*R26*^*tdT*^: *n* = 3 (2w CTL, 8w CTL, 12w CTL, 26w CTL), *n* = 4 (8w BLZ945, 12w BLZ945), *n* = 5 (26w BLZ945), *n* = 6 (5d CTL), *n* = 8 (5d BLZ945), mean ± SEM. Ordinary one-way ANOVA with Šidák-adjusted multiple-comparisons p values. (**e-f**) Representative images (**e**) and quantifications (**f**) of labeled MG, leptomeningeal (lmMΦ) and perivascular (pvMΦ) macrophages. Symbols represent individual mice, *Hexb*^*CreERT2*^*R26*^*YFP*^: *n* = 4; *Mrc1*^*CreERT2*^*R26*^*tdT*^: *n* = 4 (olfactory tubercle BLZ945), *n* = 5 (all other groups), mean ± SEM. Unpaired two-tailed t-test with Welch’s correction. Arrowheads: magenta = YFP^+^ MG; red = YPF^−^ MG; blue = YFP^−^ leptomeningeal (lmMΦ) and perivascular (pvMΦ) macrophages; open = tdT^+^ lmMΦ and pvMΦ; orange = tdT^−^ lmMΦ and pvM. (**g**) Representative images from *Cxcr4*^*CreERT2*^*R26*^*tdT*^ mice. Arrowheads: open = tdT^−^ lmMΦ and pvMΦ; orange = tdT^+^ lmMΦ and pvMΦ. (**h-k**) Flow cytometric analysis. Representative histograms (**h**) and quantification of reporter gene expression in MG and sdCAMs from *Hexb*^*CreERT2*^*R26*^*YFP*^ (**i**), *Mrc1*^*CreERT2*^*R26*^*tdT*^ (**j**) and *Cxcr4*^*CreERT2*^*R26*^*tdT*^ (**k**). Symbols represent individual mice, *Hexb*^*CreERT2*^*R26*^*YFP*^: *n* = 3 (2w CTL), *n* = 4 (2w BLZ945, 8w, 26w); *Mrc1*^*CreERT2*^*R26*^*tdT*^: *n* = 3 (2w CTL, 26w CTL), *n* = 4 (2w BLZ945, 26w BLZ945), *n* = 6 (8w); *Cxcr4*^*CreERT2*^*R26*^*tdT*^: *n* = 3 (5d CTL, 2w CTL, 8w CTL, 12w CTL, 26w CTL), *n* = 4 (8w BLZ945, 12w BLZ945), *n* = 5 (5d BLZ945, 26w BLZ945), *n* = 6 (2w BLZ945), mean ± SEM. Ordinary one-way ANOVA with Šidák-adjusted multiple-comparisons p values. (**l**) Representative images showing Ki-67 immunofluorescence in the cortex of *Cxcr4*^*CreERT2*^*R26*^*tdT*^ mice 5d post CTL or BLZ945 treatment, respectively and quantification. Arrowheads: blue = Ki-67^−^ lmMΦ and pvMΦ; red = Ki-67^+^ lmMΦ and pvMΦ. Symbols represent individual mice, *n* = 3 per group, mean ± SEM. Unpaired two-tailed t-test with Welch’s correction (MG) or ordinary one-way ANOVA with Šidák’s multiple comparison test, multiplicity adjusted p values (lmMΦ and pvMΦ). Source data

To decipher the cellular source of replenished microglia and sdCAMs, we next took advantage of myeloid subset-specific inducible CreER lines that selectively label either microglia (*Hexb*^*CreERT2*^*R26*^*YFP*^)^18^, CAMs (*Mrc1*^*CreERT2*^*R26*^*tdT*^)^3,12^ or hematopoietic stem cell (HSC)-derived cells (*Cxcr4*^*CreERT2*^*R26*^*tdT*^)^12,39,40^ (Fig. 3a, b and Extended Data Fig. 3a). While Ly6C^hi^ or Ly6C^lo^ monocytes were not labeled in *Hexb*^*CreERT2*^*R26*^*YFP*^ and *Mrc1*^*CreERT2*^*R26*^*tdT*^ mice, these cells were efficiently labeled in *Cxcr4*^*CreERT2*^*R26*^*tdT*^ animals (Extended Data Fig. 3b–d). Notably, microglia showed consistent labeling in the cortex of *Hexb*^*CreERT2*^*R26*^*YFP*^ mice across all time points, whereas only single lmMΦ and pvMΦ were YFP^+^ (Fig. 3c, d). In contrast, labeling of renewed cortical lmMΦ and pvMΦ in *Mrc1*^*CreERT2*^*R26*^*tdT*^ mice dropped compared to controls, whereas virtually no tdT^+^ microglia were observed (Fig. 3e, f). Of note, besides some consistent labeling in nonimmune cells (Supplementary Fig. 3), brains of *Cxcr4*^*CreERT2*^*R26*^*tdT*^ animals showed substantial tdT labeling in lmMΦ and pvMΦ after depletion, but not in microglia (Fig. 3g, h and Extended Data Fig. 3e–k). After depletion, both resident yolk sac (YS)- as well as HSC-derived sdCAMs were positive for the proliferation marker Ki-67 (Extended Data Fig. 3l). In summary, our data demonstrate cell-autonomous repopulation of microglia. In stark contrast, sdCAMs utilize both surviving remnants as well as peripheral HSC-derived cells to refill their niches in a heteronomous manner.

## Adhesion molecules shape transient infiltration of monocytes

**Fig. 4:**
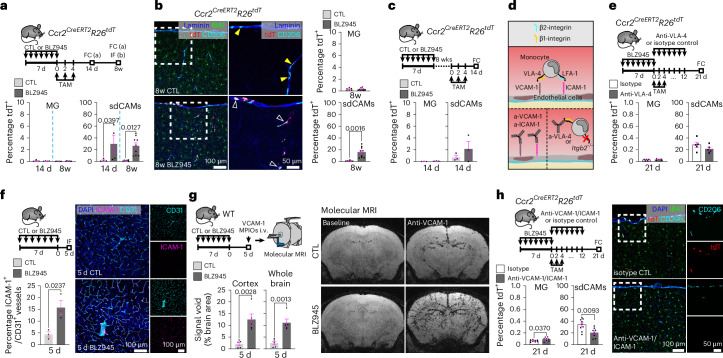
Monocytic infiltration into CNS interfaces is transient and dependent on adhesion molecules. **a**,**b**, Experimental scheme (top). Recombination rates in MG and sdCAMs were determined by flow cytometry (FC) (**a**) or immunofluorescence (IF) (**b**). Typical immunofluorescence pictures of cortices from *Ccr2*^*CreERT2*^*R26*^*tdT*^ mice 8w after treatment and quantification of IBA1^+^tdT^+^ MG and CD206^+^tdT^+^ CAMs. Arrowheads, yellow show CD206^+^tdT^−^ CAMs; blank show CD206^+^tdT^+^ CAMs. Symbols represent individual mice, *n* = 4 (14 d), *n* = 6 (8w CTL), *n* = 9 (8w BLZ945), mean ± s.e.m. Ordinary one-way ANOVA with Šidák-adjusted multiple-comparisons *P* values. **c**, Experimental scheme (top). FC quantification of tdT^+^ MG and sdCAMs. Symbols represent individual mice, *n* = 3 per group, mean ± s.e.m. Ordinary one-way ANOVA with Šidák-adjusted multiple-comparisons *P* values. **d**, Diagram of integrins and their receptors in cellular extravasation. **e**, Experimental scheme (top). FC quantification of tdT^+^ MG or sdCAMs. *n* = 5 per group. Symbols represent individual mice, mean ± s.e.m. Two-tailed *t*-test. **f**, Experimental scheme (top left). Representative pictures of anti-ICAM-1 immunofluorescence (right) and quantification of ICAM-1^+^CD31^+^ cerebral cortex vasculature 5 d after treatment (bottom left). Symbols represent individual mice, *n* = 3 per group, mean ± s.e.m. Unpaired two-tailed *t*-test. **g**, Experimental scheme (top left). Representative MRI images (right) and quantification thereof (bottom left). Symbols represent individual mice, *n* = 5 per group, mean ± s.e.m. Ordinary one-way ANOVA with Šidák-adjusted multiple-comparisons *P* values. MPIO, microparticles of iron oxide. **h**, Experimental scheme (top left). FC quantification of tdT^+^ MG or CAMs (bottom left). Typical immunofluorescence pictures of cortices from *Ccr2*^*CreERT2*^*R26*^*tdT*^ mice 21 d after treatment (right). *n* = 9 (isotype), *n* = 8 (anti-VCAM-1/ICAM-1). Symbols represent individual mice, mean ± s.e.m. Unpaired two-tailed *t*-test. Source data

**Extended Data Fig. 4:**
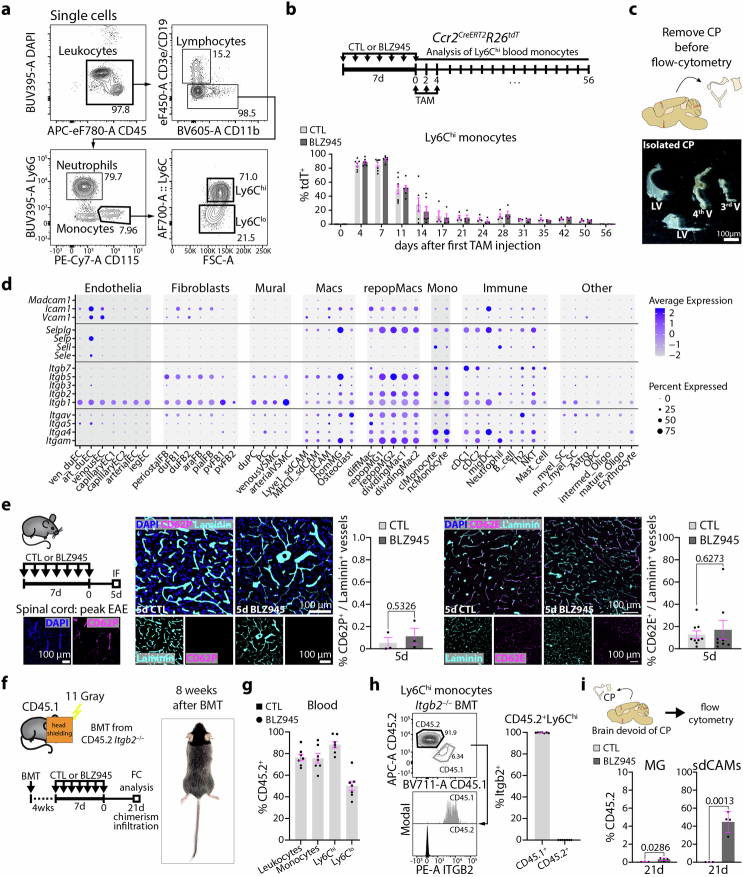
Modulation of monocyte infiltration into CNS interfaces by integrins. (**a**) Representative gating strategy for blood. (**b**) Experimental scheme and quantification of tdTomato (tdT) reporter expression. Symbols represent individual mice, *n* = 4 (17d–56d), *n* = 6 (11d BLZ945, 14d), *n* = 8 (11d CTL, 0d–7d), mean ± SEM. (**c**) Representative images of isolated choroid plexus (CP) from lateral ventricles (LV), 3^rd^ ventricle (V) or 4^th^ V. (**d**) Gene expression of selected cell adhesion molecules and integrins in leptomeninges and cortex (related to Fig. 1). Color scale: Relative expression levels. (**e**) Experimental scheme, representative images and quantification of CD62P^+^Laminin^+^ or CD62E^+^Laminin^+^ cerebral cortex vasculature. Symbols represent individual mice, *n* = 3 (CD62P), *n* = 8 (CD64E BLZ945), *n* = 9 (CD64E CTL), mean ± SEM, unpaired two-tailed t-test. Positive control for CD62P: Spinal cord of an experimental autoimmune encephalomyelitis (EAE) mouse at peak (Score 3). (**f-i**) Experimental scheme (left) and representative photography of a mouse 8 weeks after whole bone marrow (BMT) transplantation (right) (**f**). Flow cytometric quantification of CD45.2^+^ immune cells (**g**) and validation of successful ITGB2 knock-out in CD45.2^+^ Ly6C^hi^ monocytes (**h**) in recipient blood at 21d. Flow cytometric quantification of CD45.2^+^ microglia (MG) and sdCAMs (**i**). Symbols represent individual mice, *n* = 3 (BLZ945), *n* = 4 (CTL), mice pooled for figure (**h**) Mean ± SEM, unpaired two-tailed t-test in (**i**). CP: Choroid plexus; FC: Flow cytometry. Source data

Having identified descendants of CXCR4^+^ HSCs as a partial source of replenished sdCAMs, we next aimed to uncover their direct progenitor. As Ly6C^hi^CCR2^hi^ monocytes are known to differentiate into brain macrophages upon perturbation^23,41^, we labeled them during the first two weeks of repopulation using *Ccr2*^*CreERT2*^*R26*^*tdT*^ mice (Fig. 4a and Extended Data Fig. 4a–c). We detected a significant increase of replenished tdT^+^CD206^+^ sdCAMs, whereas microglia consistently remained unlabeled (Fig. 4a, b). Conversely, labeling Ly6C^hi^CCR2^hi^ monocytes 8w post-depletion, when microglia and sdCAM numbers were fully restored, did not lead to an increase in tdT^+^ sdCAM proportion (Fig. 4c), revealing only transient monocyte engraftment after niche induction.

To explore the underlying mechanisms of this monocytic influx, we first checked the gene expression of adhesion molecules and integrins guiding monocytic extravasation^42^ (Fig. 4d) in our scRNA-seq dataset (Extended Data Fig. 4d). While monocytes expressed high levels of integrin α-4 (*Itga4*) as well as *Itgb1* and *Itgb2*, CNS endothelial cells showed expression of *Icam1*, *Vcam1*, *Sele* and *Selp* (Extended Data Fig. 4d). Notably, no differential expression of the endothelial adhesion molecules P-Selectin (CD62P) and E-Selectin (CD62E) was detectable at protein level 5 d after depletion (Extended Data Fig. 4e). To test which molecules mediate monocyte infiltration, we next targeted integrin receptors. Injection of very late antigen-4 (VLA-4) neutralizing antibodies during the first two weeks of repopulation in *Ccr2*^*CreERT2*^*R26*^*tdT*^ mice, did not significantly decrease the proportion of tdT^+^ monocyte-derived sdCAMs at 21 d after depletion (Fig. 4e). Similarly, CD45.2^+^ ITGB2-deficient monocytes did not show impaired engraftment into the sdCAM compartment (Extended Data Fig. 4f–i).

Therefore, we next assessed the integrin receptors intercellular adhesion molecule 1 (ICAM-1; Fig. 4f) and vascular cell adhesion molecule 1 (VCAM-1; Fig. 4g) on brain endothelial cells. Both proteins were found to be upregulated 5 d after sdCAM depletion (Fig. 4f, g). To evaluate their functional relevance, we injected *Ccr2*^*CreERT*^*R26*^*tdT*^ mice with ICAM-1 and VCAM-1 blocking antibodies during the first 2 weeks of repopulation and found reduced amounts of tdT^+^ monocyte-derived sdCAMs (Fig. 4h). Taken together, our data show that sdCAM repopulation is partially dependent on an early but transient engraftment of circulating monocytes, which is mediated by interactions with VCAM-1/ICAM-1 on endothelial cells.

## dCAMs repopulate through amplified monocyte infiltration

**Extended Data Fig. 5:**
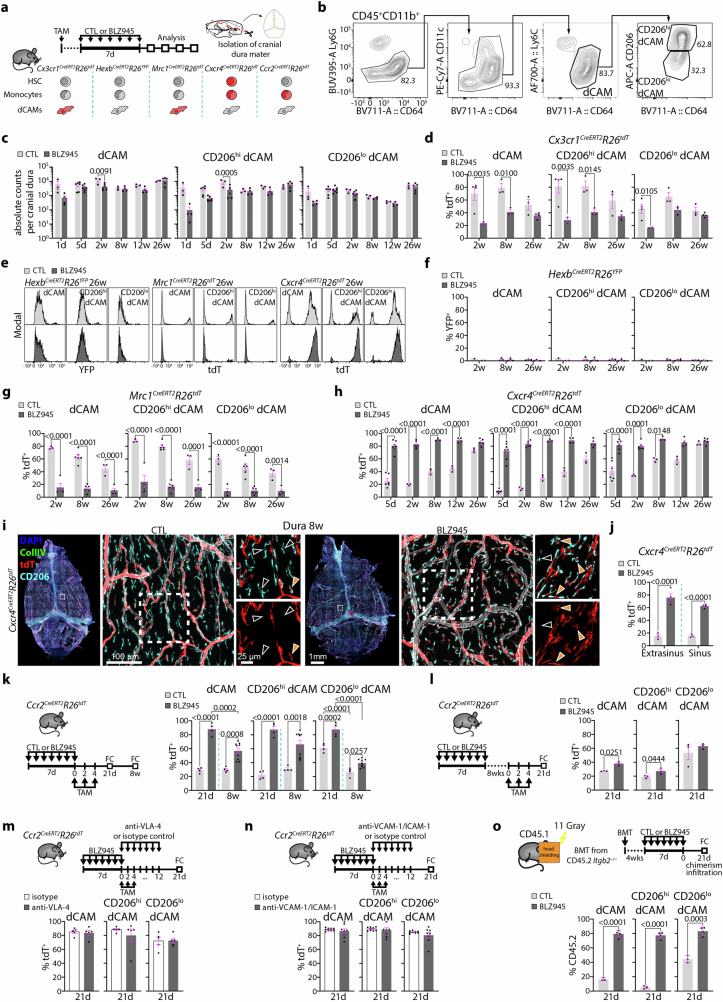
Repopulation of macrophages in the dura mater. (**a**) Experimental scheme. CTL: control; HSC: hematopoietic stem cell; Mono: monocyte; Tamoxifen: TAM (TAM); dCAMs: dural CAMs. (**b**) Representative gating strategy. (**c**) dCAM count per cranial dura mater quantified by flow cytometry. Symbols represent individual mice, *n* = 3 (1d, 2w, 8w, 12W, 26w all CTL), *n* = 4 (1d BLZ945, 8w BLZ945, 12w BLZ945), *n* = 5 (26w BLZ945), *n* = 6 (5d CTL, 2w BLZ945), *n* = 7 (5d BLZ945), mean ± SEM. Ordinary one-way ANOVA with Šidák-adjusted multiple-comparisons p values. (**d**) Flow cytometry-based quantification of tdTomato^+^ (tdT^+^) dCAMs from *Cx3cr1*^*CreERT2*^*R26*^*tdT*^ mice. Symbols represent individual mice, *n* = 2 (2w BLZ945), *n* = 3 (8w, 26w CTL), *n* = 4 (2w CTL, 26w BLZ945), mean ± SEM. Ordinary one-way ANOVA with Šidák-adjusted multiple-comparisons p values. (**e-h**) Flow cytometric analysis. Representative histograms (**e**) and quantification of reporter gene expression in dCAMs from *Hexb*^*CreERT2*^*R26*^*YFP*^ (**f**), *Mrc1*^*CreERT2*^*R26*^*tdT*^ (**g**) and *Cxcr4*^*CreERT2*^*R26*^*tdT*^ (**h**) mice. Symbols represent individual mice, *Hexb*^*CreERT2*^*R26*^*YFP*^: *n* = 1 (2w CTL), *n* = 2 (2w BLZ945), *n* = 3 (8w CTL, 26w CTL), *n* = 4 (8w BLZ945, 26w BLZ945), *Mrc1*^*CreERT2*^*R26*^*tdT*^: *n* = 3 (2w CTL, 26w CTL), *n* = 4 (2w BLZ945, 26w BLZ945), *n* = 6 (8w), *Cxcr4*^*CreERT2*^*R26*^*tdT*^: *n* = 3 (2w, 8w, 12w, 26w all CTL), *n* = 4 (8w BLZ945, 12w BLZ945), *n* = 5 (26w BLZ945), *n* = 6 (5d CTL, 2w BLZ945), *n* = 7 (5d BLZ945), mean ± SEM. Ordinary one-way ANOVA with Šidák-adjusted multiple-comparisons p values. (**i, j**) Representative images (**i**) and histological quantification (**j**) of tdT^+^ dCAMs in extrasinus and sinus area of cranial dura mater. Arrowheads: open = tdT^−^ dCAMs; orange = tdT^−^ dCAMs. Symbols represent individual mice, *n* = 3 (CTL), *n* = 4 (BLZ945), mean ± SEM. Ordinary one-way ANOVA with Tukey HSD-adjusted multiple-comparisons p values. (**k-n**) Experimental schemes and quantification of tdT^+^ dCAMs by flow cytometry (FC). Symbols represent individual mice; **k**: *n* = 4 (21d, 8w CTL), *n* = 9 (8w BLZ945); **l**: *n* = 3 per group; **m**: *n* = 5 per group; **n**: *n* = 9 (CTL), *n* = 8 (BLZ945); mean ± SEM. Ordinary one-way ANOVA with Tukey’s multiple comparison test, multiplicity adjusted p values (**k**) or Unpaired two-tailed t-test (**l, m, n**). (**o**) Experimental scheme and quantification CD45.2^+^ dCAMs by FC. Symbols represent individual mice, *n* = 3 (CTL), *n* = 4 (BLZ945), mean ± SEM. Unpaired two-tailed t-test.

In contrast to sdCAM niches and the CNS parenchyma, the healthy dura mater is readily accessible to monocytes, making dCAMs a heterogenous population of mostly HSC origin^3,29^. We therefore compared the repopulation kinetics and cellular origin of dCAMs to those observed for sdCAMs and microglia (Extended Data Fig. 5a, b). While dCAMs were strongly reduced at 1 d after depletion, they reached normal numbers by 5 d (Extended Data Fig. 5c). Analysis of their cellular origin under control conditions clearly revealed continuous exchange of dCAMs by HSC-derived cells, with CD206^lo^ dCAMs showing a higher rate of homeostatic exchange compared to CD206^hi^ dCAMs (Extended Data Fig. 5d–h). After depletion, both CD206^hi^ and CD206^lo^ dCAMs were replaced to similar near-complete rates, exceeding those seen in sdCAMs (Extended Data Fig. 5d–h). We did not detect significant differences in the amount of monocyte-derived dCAMs between extrasinusoidal and sinusoidal compartments^3^ 8w after depletion (Extended Data Fig. 5i, j). We found a comparable ratio of tdT^+^ dCAMs in both *Ccr2*^*CreERT2*^*R26*^*tdT*^ and *Cxcr4*^*CreERT2*^*R26*^*tdT*^ mice, suggesting that repopulated dCAMs are mostly derived from monocytes (Extended Data Fig. 5k, l). In line with our observations for sdCAMs, there was no reduction of monocyte-derived dCAMs following injection of VLA-4-neutralizing antibodies or after transplantation of CD45.2^+^*Itgb2*^−/−^ bone marrow. In contrast to sdCAMs, injection of anti-VCAM-1 and anti-ICAM-1 antibodies did not affect the infiltration of monocyte-derived dCAMs (Extended Data Fig. 5m–o). Therefore, dCAMs repopulate rapidly through an enhancement of physiological monocyte engraftment.

## Long-term transcriptional alterations in repopulated sdCAMs

**Fig. 5:**
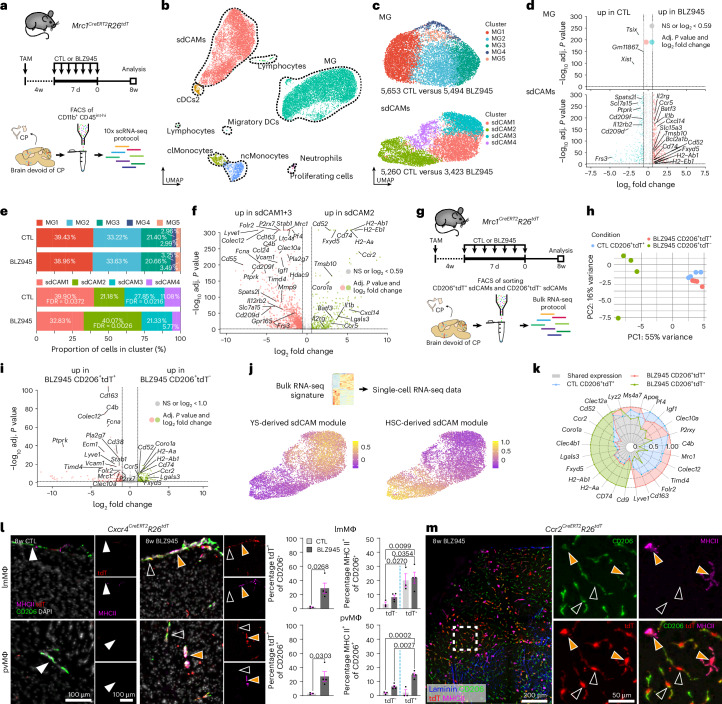
Long-term transcriptional changes in sdCAMs but not MG after repopulation. **a**, Experimental scheme. TAM, tamoxifen; CP, choroid plexus. *n* = 4 mice per treatment. **b**, Annotated UMAP of 21,402 individual cells. **c**, Reclustered UMAPs of 11,147 MG and 8,683 sdCAMs. **d**, Volcano plots comparing MG and sdCAMs from 8w after BLZ945 to CTL. Bonferroni-corrected *P* values. NS, not significant, *P* > 0.05. **e**, Cluster proportions for MG or sdCAMs at 8w after CTL or BLZ945 treatment. *n* = 4 mice per treatment, arithmetic mean across biological replicates, arcsine transformation with *t*-test and Benjamini–Hochberg false discovery rate (FDR) correction. **f**, Volcano plot comparing DEGs between sdCAM2 to sdCAM1 and sdCAM3 (**c**). Bonferroni-corrected *P* values, NS: *P* > 0.05. **g**,**h**, Experimental scheme. CPs were removed and CD206^+^tdT^+^ and CD206^+^tdT^−^ sdCAMs were sorted for bulk RNA-seq (**g**). PCA (**h**). *n* = 4 mice per treatment. **i**, Volcano plot depicting DEGs between CD206^+^tdT^+^ sdCAMs and CD206^+^tdT^−^ sdCAMs. Benjamini–Hochberg-adjusted *P* values. NS, *P* > 0.05. **j**, Genes found to be upregulated in CD206^+^tdT^+^ sdCAMs or CD206^+^tdT^−^ sdCAMs in the bulk RNA-seq dataset (**i**) were plotted onto the scRNA-seq sdCAM dataset (**c**) as module scores for feature plots. **k**, Radar chart depicting relative expression levels of selected genes across sdCAM populations from bulk RNA-seq. Per gene, highest expression level is scaled to 1. **l**, Immunofluorescence based quantification of MHC class II expression in brains of *Cxcr4*^*CreERT2*^*R26*^*tdT*^ mice. Arrowheads, white show MHC II^−^CD206^+^tdT^−^ cell; blank show MHC II^−^CD206^+^ tdT^+^ cell; orange show MHC II^+^CD206^+^tdT^+^ cell. Symbols represent individual mice, *n* = 3 (CTL), *n* = 4 (BLZ945), mean ± s.e.m. Ordinary one-way ANOVA with Šidák-adjusted multiple-comparisons *P* values. DAPI, 4,6-diamidino-2-phenylindole. **m**, Representative immunofluorescence image of MHC II expression in whole mount leptomeninges of *Ccr2*^*CreERT2*^*R26*^*tdT*^ mice. Arrowheads, blank show MHC II^−^CD206^+^tdT^+^ cell; orange show MHC II^+^CD206^+^tdT^+^ cell. Representative of three mice. Source data

**Extended Data Fig. 6:**
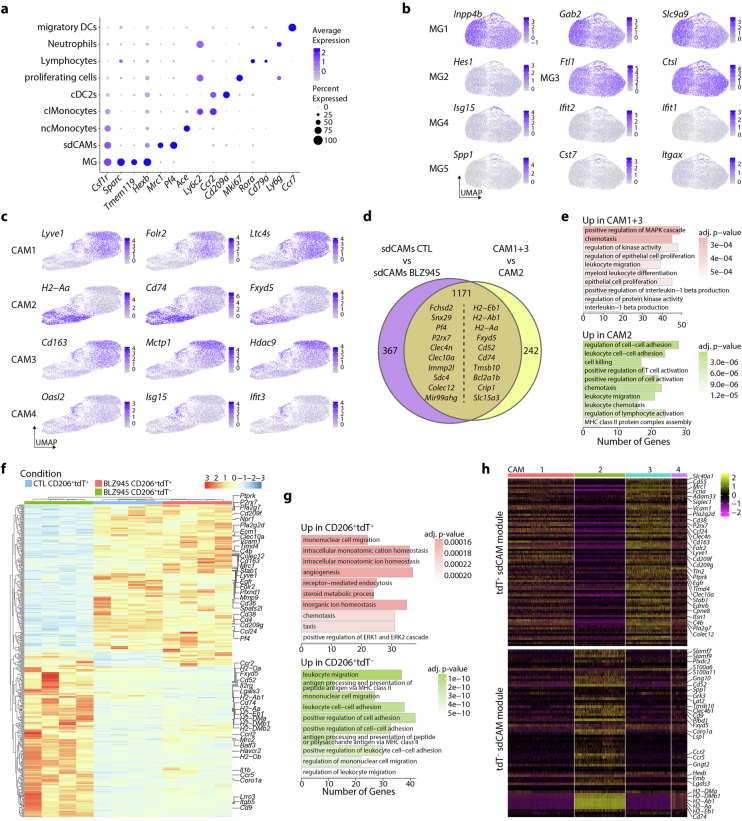
Cluster markers of microglia and sdCAMs. Dot plot depicting selected cell-type-defining markers. Color scale: Relative expression levels. (**b, c**) Feature plots of selected cluster defining markers for MG clusters 1–5 (MG1-5) (**b**) and sdCAM clusters 1–4 (sdCAM1-4) (**c**) (Related to Fig. 5c). Scale bars show z-scores of minimum to maximum scaled gene expression. (**d**) Venn diagram depicting overlay between DEGs (Bonferroni adjusted *p* < 0.05 and |log2 fold change| > 0.59) between all sdCAMs from BLZ945 vs CTL mice and DEGs between CAM1 and 3 vs CAM2. Top 10 overlapping up (left) and down (right) regulated genes are shown. (**e**) Gene ontology (GO) term analysis showing upregulated terms from sdCAM1 and 3 compared to sdCAM2. p values are Benjamini–Hochberg adjusted. (**f**) Heatmap depicting the top 472 DEGs (Benjamini–Hochberg adjusted *p* < 0.05 and |log2 fold change| > 1) from the bulk RNA-seq analysis (Related to Fig. 5g). *n* = 4 mice per group, scale bar indicates z-score normalized expression. (**g**) GO term analysis of CD206^+^tdT^+^ CAMs and CD206^+^tdT^−^ CAMs. p values are Benjamini–Hochberg adjusted. (**h**) Genes found to be upregulated in either CD206^+^tdT^+^ CAMs or CD206^+^tdT^−^ CAMs in the bulk RNA-seq dataset (Fig. 5i) were provided as input to the scRNA-seq CAM dataset to generate cluster-wise heatmaps.

To characterize comprehensively the replenished microglia and sdCAMs at the molecular level, we performed scRNA-seq at 8 weeks post-depletion (Fig. 5a and Supplementary Fig. 4a). A total of 21,402 individual immune cells were annotated according to their cell-type-specific markers^3,33^ (Fig. 5b and Extended Data Fig. 6a). Microglia were virtually unaffected by the treatment, with only three genes slightly regulated, whereas 648 differentially expressed genes (DEGs) were identified for repopulated sdCAMs (Fig. 5c). Further subclustering resulted in five microglia clusters (Fig. 5d and Supplementary Fig. 4b–d). Microglia clusters 1–4 (MG1–MG4) had a typical homeostatic signature expressing *Sparc, Hexb, P2ry12* and *Siglech*, whereas MG5 contained few cells with subtle induction of *Spp1*, *Cst7* and *Itgax* (Extended Data Fig. 6b and Supplementary Fig. 4e, f). In contrast, sdCAMs split into four distinct clusters (sdCAM1–sdCAM4) (Fig. 5d and Supplementary Fig. 4b, d) with sdCAM1, 3 and 4 displaying high expression of *Mrc1*, *Pf4* and *Lyve1*, whereas sdCAM2 instead expressed higher levels of MHC class II-related genes such as *H2-Aa* and *Cd74* (Extended Data Fig. 6c and Supplementary Fig. 4g, h). Of note, all sdCAM and microglia clusters were found in control and repopulated mice; however, we found significant alterations in the composition of repopulated sdCAMs, but not in microglia. After repopulation, sdCAM2 made up 40.07% of sdCAMs (21.18% in control), accompanied by a significant decrease of sdCAM1 to 32.83% (39.90% in control) and sdCAM3 to 21.33% (27.85% in control) (Fig. 5e). DEG analysis between the affected sdCAM clusters showed increased expression levels of MHC class II-related genes, *Ccr2* and *Fxyd5* in sdCAM2, whereas *Lyve1*, *Folr2* and *Cd163* were up in sdCAM1 + 3 (Fig. 5f and Extended Data Fig. 6d, e). In summary, microglia regained their initial transcriptional profile after repopulation, whereas sdCAM composition remained altered, shifting toward the immunologically primed sdCAM2 phenotype.

## Ontogeny drives phenotypic changes in repopulated sdCAMs

As scRNA-seq revealed long-term transcriptional changes only in sdCAMs, we next investigated how developmental origin contributes to sdCAM phenotypes after repopulation. We thus performed bulk RNA-seq of YS-derived CD206^+^tdT^+^ and HSC-derived CD206^+^tdT^−^ sdCAMs from *Mrc1*^*CreERT2*^*R26*^*tdT*^ mice 8w after depletion (Fig. 5g). Principal-component analysis (PCA) showed a close association of control tdT^+^ sdCAMs with re-emerged tdT^+^ sdCAMs, whereas renewed tdT^−^ sdCAMs were clearly distinct (Fig. 5h). Accordingly, DEG analysis revealed only minor differences between tdT^+^ sdCAMs from repopulated and control mice, but major changes compared to tdT^−^ sdCAMs (Fig. 5i and Extended Data Fig. 6f). tdT^+^ sdCAMs highly expressed the typical sdCAM markers *Cd163*, *Lyve1* and *C4b*, whereas tdT^−^ sdCAMs showed stronger expression of MHC class II-related genes as well as *Fxyd5* and *Ccr2* (Extended Data Fig. 6f). The differences closely resembled those identified between sdCAM1 + 3 and sdCAM2 in the scRNA-seq analysis (Fig. 5a–f). Gene Ontology (GO) term analysis revealed enrichment of a ‘chemotactic’ phenotype in tdT^+^ sdCAMs and a ‘leukocyte’ and ‘regulation of cell adhesion’ pattern in HSC-derived tdT^−^ sdCAMs (Extended Data Fig. 6g). We then investigated whether sdCAM phenotypes found in our bulk RNA-seq dataset would align with sdCAM clusters detected by scRNA-seq (Fig. 5a–f). Indeed, the genes upregulated in tdT^+^ sdCAMs were highly and selectively expressed in sdCAM1 and sdCAM3, whereas the genes upregulated in tdT^−^ sdCAMs were nearly solely found in sdCAM2 (Fig. 5j and Extended Data Fig. 6h). Assessment of the most consistent genes from scRNA-seq and bulk RNA-seq established a common sdCAM gene signature (*Clec12a*, *Lyz2*, *Ms4a7* and *Apoe*) as well as unique signatures for YS-derived tdT^+^ and HSC-derived tdT^−^ sdCAMs (Fig. 5k).

In *Cxcr4*^*CreERT2*^*R26*^*tdT*^ fate mapping mice, HSC-derived CD206^+^tdT^+^ sdCAMs were found in the leptomeninges and perivascular space in repopulated animals. Consistent with the observed transcriptomic changes, they were frequently positive for MHC class II (Fig. 5l). In addition, replenished CD206^+^tdT^+^ lmMΦ coexpressed MHC class II in repopulated *Ccr2*^*CreERT2*^*R26*^*tdT*^ mice (Fig. 5m). In summary, during repopulation, HSC-derived sdCAMs preferentially gain an MHC II^hi^ phenotype and thus shape the CNS border macrophage transcriptome long-lastingly.

## Ontogeny and repopulation-associated chromatin patterns

**Fig. 6:**
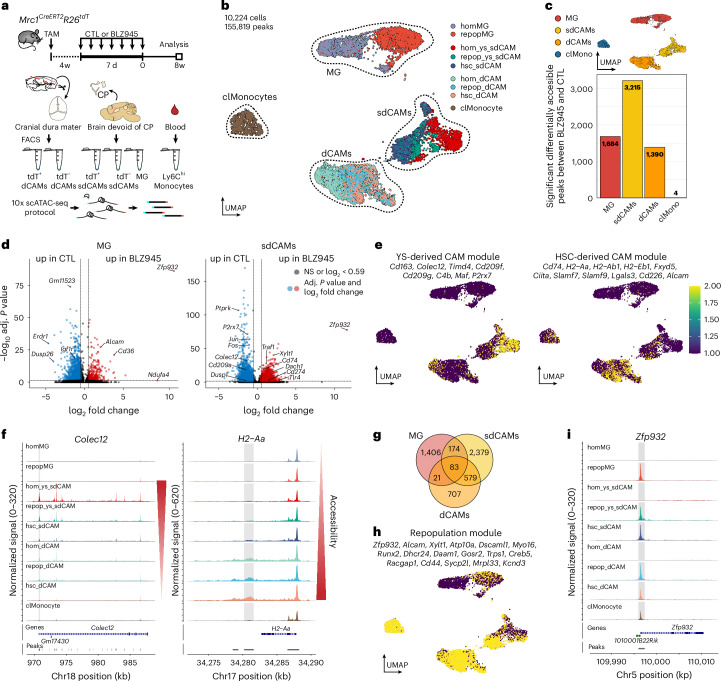
Cellular ontogeny shapes chromatin accessibility in CNS macrophages. **a**, Experimental scheme. *Mrc1*^*CreERT2*^*R26*^*tdT*^ mice were injected with TAM and either treated with BLZ945 or CTL. CPs were removed and MG, CD206^+^tdT^+^ and CD206^+^tdT^−^ sdCAMs, CD206^+^tdT^+^ and CD206^+^tdT^−^ dCAMs as well as Ly6C^hi^ blood monocytes were sorted for scATAC-seq. Cells pooled from *n* = 3 (CTL) and *n* = 4 (BLZ945) mice. **b**, UMAP based on called peaks of 10,224 individual nuclei with cell type annotation. **c**, Bar graph depicting all significant differentially accessible peaks between BLZ945 and CTL for each cell type. **d**, Volcano plots comparing accessible peaks in MG and sdCAMs from 8w after BLZ945 to CTL. Bonferroni-corrected *P* values. NS, *P* > 0.05. **e**, Feature plots depicting module scores derived from selected differential gene activities between hom_ys_sdCAM and hsc_sdCAM. **f**, Coverage plots of *Colec12* and *H2-Aa* loci depicting the aggregated ATAC reads for each cell subtype. Differentially accessible peaks are highlighted in gray. **g**, Venn diagram showing overlap of differentially accessible peaks in the BLZ945 compared to CTL condition for the depicted cell types. **h**,**i**, Feature plot displaying a repopulation module score calculated from differential gene activities that are commonly and significantly enriched in macrophages from BLZ945-treated animals compared to CTL (**i**). Coverage plot of the *Zfp932* locus depicting the aggregated ATAC reads for each cell cluster. Differentially accessible peak is highlighted in gray.

**Extended Data Fig. 7:**
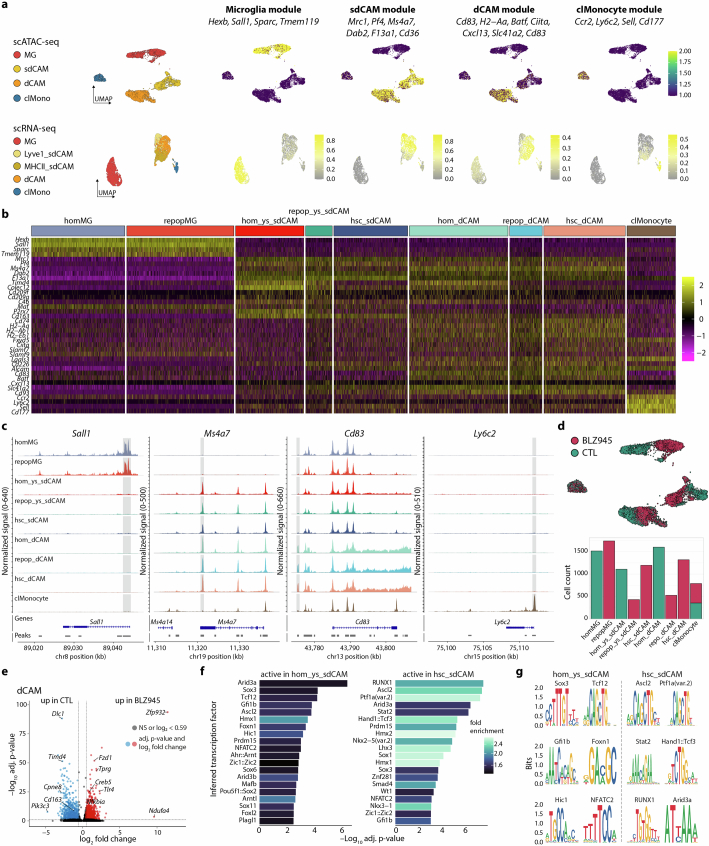
scATAC-seq of repopulated CNS macrophages. (**a**) Feature plots displaying module scores calculated from gene activities for the distinct cell populations based on scATAC-seq (top) or for the same genes based on gene expression levels derived from scRNA-seq from Fig. 2 (bottom). Scale bars show z-scores of minimum to maximum scaled gene activity (top) or gene expression (bottom). (**b**) Heatmap depicting gene activities of identified scATAC-seq marker genes for CNS macrophage populations. Scale bar shows z-score of gene activity. (**c**) Coverage plots of *Sall1, Ms4a7, Cd83* and *Ly6c2* loci depicting the aggregated ATAC reads for each cell cluster. Differentially accessible peaks are highlighted in gray. (**d**) Feature plot overlaying the treatment (top) and bar plot depicting the number of nuclei derived from either of the two treatments within each cell cluster (bottom). (**e**) Volcano plots comparing accessible peaks in dCAMs from 8 weeks (8w) post BLZ945 to control (CTL). Bonferroni-corrected p values, NS: *p* > 0.05. (**f**) Bar plots showing inferred transcription factor activities in hom_ysCAM and hscCAM computed from chromVAR deviation z-scores using accessible transcription factor motifs. Scale bar shows fold enrichment over accessibility of respective motifs in all cells. p values are Benjamini–Hochberg adjusted. (**g**) Position weight matrix logos of enriched transcription factor motifs for hom_ysCAM and hscCAM. The height of each nucleotide letter is proportional to its frequency at that position, reflecting sequence conservation.

As engrafted HSC-derived sdCAMs showed a distinct transcriptomic pattern compared to YS-derived sdCAMs, we wondered to what extent this could be explained by epigenetic differences. Therefore, we performed single-cell assay for transposase-accessible chromatin-sequencing (scATAC-seq) of dCAMs, sdCAMs, microglia and Ly6C^hi^ blood monocytes from control *Mrc1*^*CreERT2*^*R26*^*tdT*^ mice or 8w post-depletion. The tdT reporter thereby allowed to distinguish CAMs based on their ontogeny (Fig. 6a). Cell subtypes were defined by transferring annotations from our scRNA-seq data (Fig. 2b) and cross-verification with our sorting strategy (Fig. 6a, b). Analysis revealed specific chromatin-accessibility profiles for microglia, sdCAMs, dCAMs and classical Ly6C^hi^ monocytes (Extended Data Fig. 7a, b). While microglia showed high peak enrichment at the *Sall1* locus, all CAM populations showed high accessibility of *Ms4a7*. All dCAMs showed enrichment at the *Cd83* locus and Ly6C^hi^ monocytes showed an enrichment at the *Ly6c2* locus. We validated the relevance of these differences in chromatin accessibility on RNA level (Extended Data Fig. 7a). In summary, these data provide a framework for the identification of CNS macrophage subtypes at the chromatin level.

As all macrophage populations displayed differences in chromatin accessibility following repopulation, we investigated the effects of treatment and ontogeny by comparing repopulated cells to control cells within each population (Extended Data Fig. 7d). Blood monocytes showed only four differentially accessible loci. In contrast, sdCAMs displayed 3,215 alterations in chromatin accessibility, about twofold more than found for microglia or dCAMs (Fig. 6c, d and Extended Data Fig. 7e). Both YS- and HSC-derived sdCAMs exhibited high accessibility at genes that were linked to their origins by RNA-seq (Figs. 6e and 5f, i). *Colec12* accessibility was increased in homeostatic and repopulated YS-derived sdCAMs, whereas *H2-Aa* accessibility was heightened in HSC-derived sdCAMs (Fig. 6f). Of note, some homeostatic dCAMs were covered by the YS-derived CAM module, whereas the majority of the population was covered by the HSC-derived CAM module (Fig. 6b, e). Next, we performed motif analysis to infer transcription factor activity in YS- versus HSC-derived sdCAMs. It revealed significant enrichment of Arid3a, Hic1 and NFATC2 motifs in YS-derived sdCAMs. Consistent with their recent emergence from monocytic progenitors, HSC-derived sdCAMs were enriched for the RUNX1 motif, a transcription factor known to interact with other lineage-defining factors during monocyte differentiation^43^ (Extended Data Fig. 7f, g).

We next compared peaks with higher accessibility in repopulated macrophages between CNS macrophage populations (Fig. 6g). Hereby, we identified a conserved repopulation-associated signature (Fig. 6h), including highly enriched accessibility to *Zfp932*, a zinc finger protein linked to repression of the differentiation associated hedgehog signaling pathway^44^ (Fig. 6d,i and Extended Data Fig. 7e). Taken together, all macrophages underwent chromatin remodeling following repopulation, with the most pronounced changes observed in sdCAMs, reflecting a switch in ontogeny during repopulation.

## Ontogeny impacts transcriptional response to endotoxemia

**Fig. 7:**
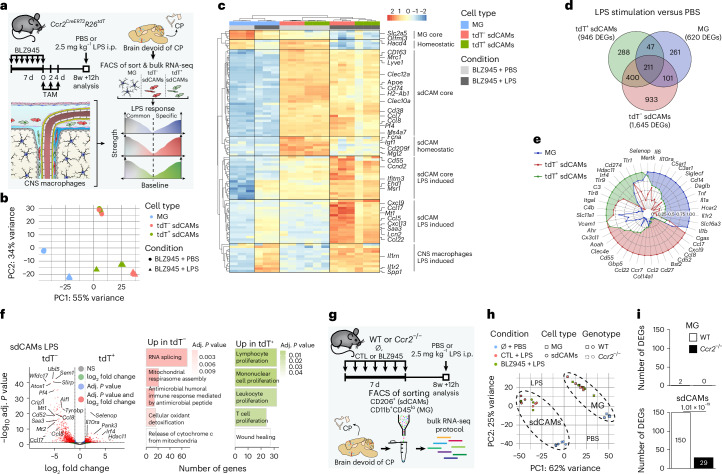
Cellular origin dictates reaction of repopulated sdCAMs to LPS challenge. **a**, Experimental scheme: Following BLZ945 treatment and tamoxifen injections, *Ccr2*^*CreERT2*^*R26*^*tdT*^ mice were intraperitoneally (i.p.) injected with PBS or LPS and MG and sdCAMs were sorted 12 h later. *n* = 3 mice per condition. **b**, PCA of MG, CD206^+^tdT^−^ sdCAMs and CD206^+^tdT^+^ sdCAMs isolated from BLZ945-treated PBS or LPS injected mice. **c**, Heatmap of the top 100 most variable genes. Scale bar indicates *z*-score normalized expression. **d**, Venn diagram depicting the overlapping and distinct number of upregulated DEGs, defined as Benjamini–Hochberg-adjusted *P* < 0.05 and |log_2_ fold change| > 1. **e**, Linear scaled radar chart depicting mean relative expression levels of selected genes within the individual CNS macrophage populations following LPS stimulation. **f**, Volcano plot showing DEGs between tdT^+^ sdCAMs and tdT^−^ sdCAMs after LPS stimulation (left). Benjamini–Hochberg-adjusted *P* values. NS, *P* > 0.05. The top five GO terms for each population are shown (right). **g**, Setup of the experiment is displayed. MG and sdCAMs were sorted from either WT or *Ccr2*^−/−^ mice that were either kept untreated (∅) or treated with CTL or BLZ945 followed by i.p. injection with PBS or LPS 12 h before termination. *n* = 3 mice per condition. **h**, PCA of MG (squares) and sdCAMs (circles) isolated from PBS or LPS injected WT and *Ccr2*^−/−^ mice. **i**, Bar plots comparing the number of DEGs, defined as Benjamini–Hochberg-adjusted *P* < 0.05 and |log_2_ fold change| > 1, for BLZ945 versus CTL between WT or *Ccr2*^−/−^ mice for MG or sdCAMs. Chi-squared test of independence for DEGs per genotype out of expressed genes.

**Extended Data Fig. 8:**
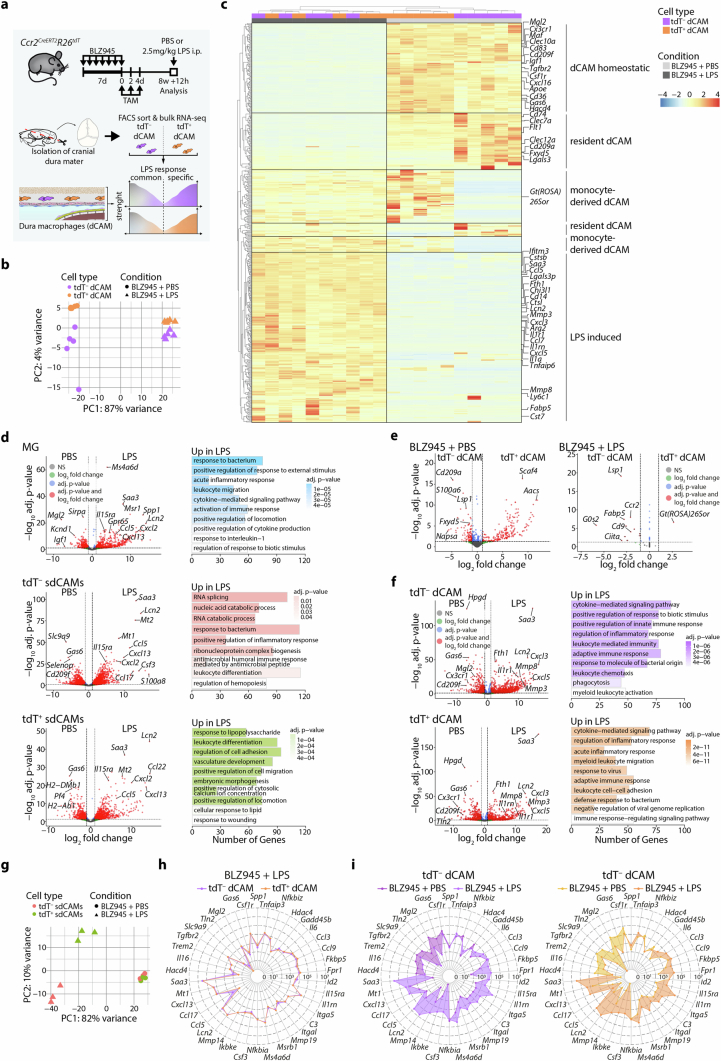
Response of repopulated CNS macrophages to LPS challenge. (**a**) Experimental overview is displayed. Following BLZ945 treatment and tamoxifen injections, *Ccr2*^*CreERT2*^*R26*^*tdT*^ mice were i.p. injected with PBS or LPS dural macrophages (dCAMs) were sorted 12h later for bulk RNA-sequencing. *n* = 5 mice per condition. (**b**) Principal-component analysis (PCA) of tdT^−^ dCAMs and tdT^+^ dCAMs isolated from BLZ945-treated PBS or LPS injected mice. (**c**) Heatmap of all 715 differentially expressed genes (DEGs; Benjamini–Hochberg adjusted *p* < 0.05 and |log2 fold change| > 1) detected between groups. *n* = 5 mice per group, scale bar indicates z-score normalized expression. (**d**) Volcano plots showing DEGs between BLZ945-treated PBS or LPS injected mice for MG, tdT^−^ sdCAMs and tdT^+^ sdCAMs. The top upregulated GO terms following LPS treatment are shown. Benjamini–Hochberg adjusted p values, NS: *p* > 0.05. (**e**) Volcano plots showing DEGs between tdT^−^ dCAMs and tdT^+^ dCAMs in BLZ945-treated PBS (left) or BLZ945 LPS injected (right) mice. Benjamini–Hochberg adjusted p values, NS: *p* > 0.05. (**f**) Volcano plots showing DEGs between BLZ945 LPS injected versus PBS injected mice for tdT^−^ dCAMs (top) and tdT^+^ dCAMs (bottom). The top upregulated GO terms following LPS treatment are shown. Benjamini–Hochberg adjusted p values, NS: *p* > 0.05. (**g**) Reclustered PCA of tdT^−^ CAMs and tdT^+^ CAMs. (**h, i**) Logarithmic scaled radar charts depicting normalized expression levels of selected genes for tdT^−^ dCAMs (top) as well as tdT^+^ dCAMs MG tdT^−^ as well as tdT^+^ dCAMs following systemic LPS challenge (**h**) and for CTL versus LPS challenge conditions for the individual populations (**i**).

After repopulation, monocyte engraftment into the sdCAM compartment generates an immunoreactive transcriptomic profile and additional chromatin-accessibility changes at the *Tlr4* locus in repopulated CAMs (Fig. 6d and Extended Data Fig. 7e). We therefore investigated cellular responses to systemic lipopolysaccharide (LPS) in CNS macrophage populations with distinct ontogeny. We exposed repopulated *Ccr2*^*CreERT2*^*R26*^*tdT*^ mice to LPS and profiled microglia, tdT^+^ and tdT^−^ sdCAMs as well as tdT^+^ and tdT^−^ dCAMs by RNA-seq 12 h after treatment (Fig. 7a, Extended Data Fig. 8a and Supplementary Fig. 5a, b). PCA and analysis of the most variable genes revealed clustering according to cell types and treatment paradigms (Fig. 7b, c and Extended Data Fig. 8b, c). Known LPS-inducible genes, including *Saa3*, *Il1rn, Spp1, Lcn2*, *Mt1*, *Ccl5* or *Ccl13*, were commonly found across all macrophage populations (Extended Data Fig. 8d–f). In line with our preceding results, the GO terms ‘leukocyte differentiation’ and ‘cell adhesion’ were enriched in HSC-derived tdT^+^ sdCAMs, whereas tdT^−^ sdCAMs showed enrichment for gene sets related to ‘RNA generation and splicing’ (Extended Data Fig. 8d). Comparison of DEGs across macrophage populations revealed partial overlap of induced genes (*n* = 211), including *Saa3*, *Lcn2*, *Ccl5* and *Cxcl13* (Supplementary Fig. 5c–e), alongside population-specific responses (Fig. 7d, e).

Focusing on sdCAM responses, tdT^−^ and tdT^+^ sdCAMs showed clear separation following LPS stimulation (Extended Data Fig. 8g). tdT^+^ sdCAMs were enriched for *Il10ra, Irf4* and *Hdac11*, resulting in the GO terms ‘proliferation of immune cell subsets’ and ‘wound healing’. In contrast, tdT^−^ sdCAMs expressed numerous inflammatory and activation-related genes such as *Aif1, Wfdc17, Saa3*, *Atox1, Mt1/2, Slirp, Sem1* or *Ubl5*, indicating ‘major cellular activation’ and ‘cell stress’ (Fig. 7f). Notably, sdCAM subsets diverged while dCAMs converged upon LPS stimulation (Extended Data Fig. 8b, g). This effect was also apparent when comparing common LPS-induced genes, which revealed no expression difference between tdT^+^ and tdT^−^ dCAMs (Extended Data Fig. 8h, i).

**Extended Data Fig. 9:**
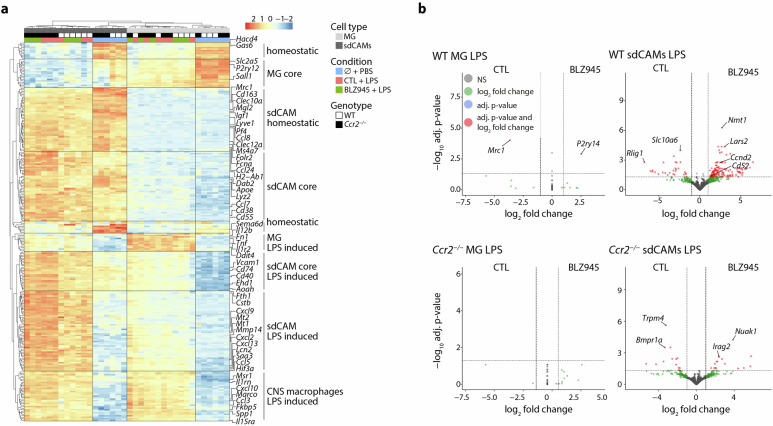
*Ccr2* deficiency prevents differential responsiveness of repopulated sdCAMs. (**a**) Heatmap of the top 250 most variable genes. Scale bar indicates z-score normalized expression. (**b**) Volcano plots showing differentially expressed genes (DEGs) between MG or sdCAMs isolated from CTL or BLZ945-treated mice following LPS treatment, respectively. Benjamini–Hochberg adjusted p values, NS: *p* > 0.05.

To determine whether HSC-derived sdCAMs alter overall sdCAM LPS responses, we applied the same model in either WT or in *Ccr2-*deficient (*Ccr2*^−/−^) mice, which lack circulating monocytes impairing the establishment of repopulated HSC-derived sdCAMs (Fig. 7g). Microglia and sdCAM samples segregated after LPS exposure, independent of the treatment group and genotype (Fig. 7h). The top 250 most variable genes revealed common as well as unique modules of regulated genes between microglia and sdCAMs (Extended Data Fig. 9a). *Msr1*, *Saa3*, *Ccl5*, *Il1rn*, *Il15ra*, *Spp1*, *Il1a/b* and *Tnfaip3* were found among the commonly upregulated genes across CNS macrophage moieties. While *Tnf* and *Il12b* were increased in microglia, *Saa3*, *Lcn2*, *Cxcl13* and *Ccl5* were more strongly induced in sdCAMs (Extended Data Fig. 9a and Supplementary Fig. 5f–i). Independent of the genotype, repopulated microglia showed an almost identical LPS response compared to controls, indicating that their reaction was not altered by depletion and repopulation (Fig. 7i and Extended Data Fig. 9b). In contrast, the LPS response of sdCAMs differed between repopulated and control WT animals, with those differences largely disappearing between repopulated and control *Ccr2*^−/−^ animals (Fig. 7i and Extended Data Fig. 9b).

Collectively, these results reveal compartment- and origin-specific signatures of CNS macrophages upon systemic immunological challenge. Monocyte engraftment into the sdCAM compartment shapes the functional response of CNS border macrophages to endotoxin, while leaving microglia unaffected in phenotype and immune responsiveness.

## Selective sdCAM replacement shapes ischemic stroke outcome

Recent evidence highlights sdCAMs as key modulators of neuroinflammation and tissue repair after stroke^45–47^, yet the impact of repopulation and replacement on these functions remains unclear. To further validate potential differences between YS- and HSC-derived sdCAMs, we examined their roles in cerebral ischemia, the second-leading cause of death and primary driver of neurological disabilities in humans.

**Fig. 8:**
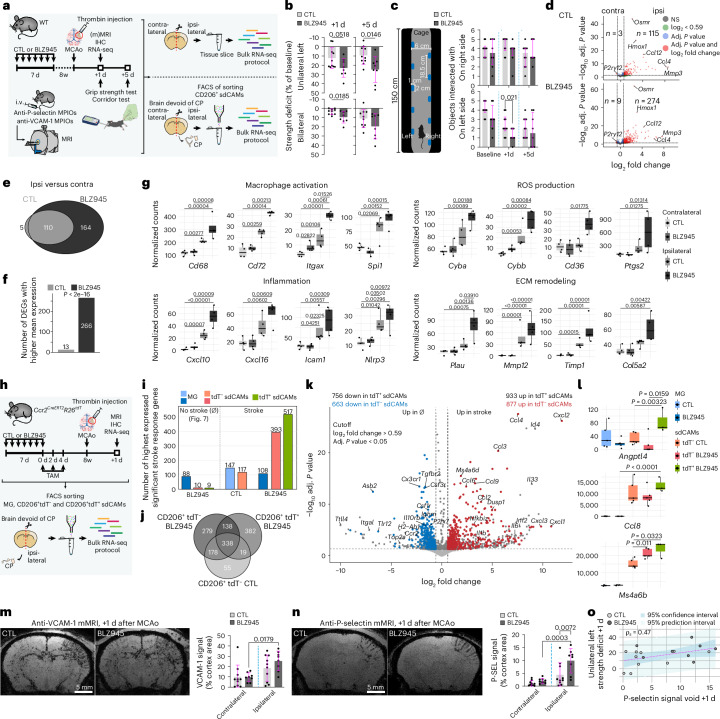
Selective replacement of sdCAMs impacts immune response and neurological outcome in ischemic stroke. **a**, Experimental scheme. IHC, immunohistochemistry; i.v., intravenous; (m)MRI, (molecular) magnetic resonance imaging; MPIO, Superparamagnetic iron oxide microparticles. **b**,**c**, Quantifications of strength deficit (**b**) and corridor test (**c**). Symbols represent individual mice, *n* = 10 (CTL), *n* = 8 (BLZ945). Mean ± s.e.m. with unpaired two-tailed *t*-test (**b**). Median ± interquartile range with two-sided Brunner–Munzel test with Benjamini–Hochberg FDR adjustment (**c**). **d**, DEGs between ipsilateral and contralateral tissue slices. *n* = 4 biological replicates per group. Benjamini–Hochberg-adjusted *P* values. NS, *P* > 0.05. **e**,**f**, Overlap (**e**) and number of stroke response genes with higher mean expression in either CTL or BLZ945 (**f**). Chi-squared test of goodness of fit. **g**, Expression levels of selected stroke response genes. *n* = 4 biological replicates per group. Ordinary one-way ANOVA with Tukey’s honest significant difference (HSD) test. **h**, Experimental scheme. *n* = 5 mice (CTL), *n* = 5 biological replicates (BLZ945, pooled from two mice each). **i**,**j**, Number of stroke response genes with higher mean expression in MG and sdCAMs from no stroke (∅) or stroke mice (**i**) and overlap of stroke response genes (**j**). Benjamini–Hochberg-adjusted *P* values, *P* < 0.05, log_2_fold change > 0.59. **k**, DEGs between tdT^+^ sdCAMs from stroke versus ∅ mice overlayed with DEGs between tdT^−^ sdCAMs from stroke versus ∅ mice. Benjamini–Hochberg-adjusted *P* values. **l**, Selection of significant stroke response genes. Symbols represent individual biological replicates, *n* = 3 (tdT^+^ sdCAMs BLZ945), *n* = 5 (all other groups). Ordinary one-way ANOVA with Tukey’s HSD test. **m**,**n**, Representative mMRI of anti-VCAM-1 (**m**) or anti-P-selectin (**n**) MPIOs and quantification. Symbols represent individual mice, *n* = 10 (CTL), *n* = 8 (BLZ945), mean ± s.e.m. Ordinary one-way ANOVA with Šidák-adjusted multiple-comparisons *P* values. **o**, Correlation between P-selectin mMRI signal and grip strength deficit. Confidence and prediction intervals are shown. *P*_S_, Spearman correlation coefficient. Source data

**Extended Data Fig. 10:**
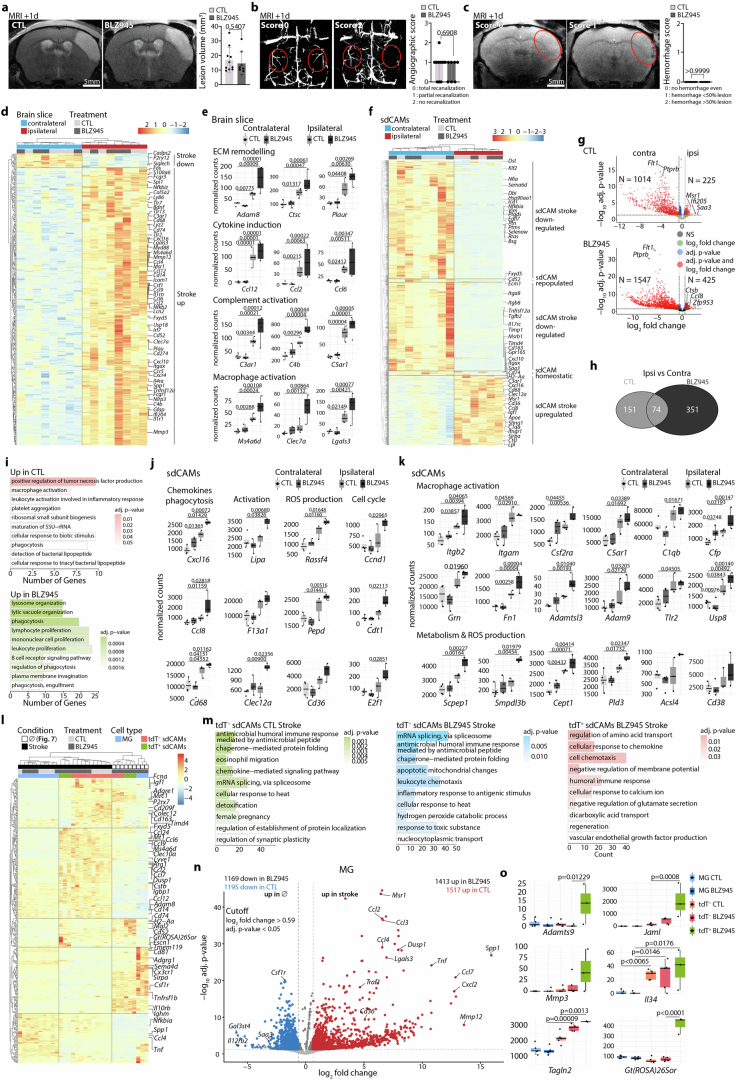
sdCAM replacement influences stroke without affecting structural characteristics. (**a-c**) Representative magnetic resonance imaging (MRI) one day after (+1d) MCAO. Quantification of lesion volume (**a**), angiographic score (**b**) and hemorrhage score (**c**). Symbols represent individual mice, *n* = 10 (CTL), *n* = 8 (BLZ945). Mean ± SEM and unpaired two-tailed t-test (**a**). Median ± interquartile range, two-tailed Mann-Whitney test (**b, c**). (**d, e**) Heatmap of all 353 differentially expressed genes (DEGs; Benjamini–Hochberg adjusted *p* < 0.05 and |log2 fold change| > 0.59) between ipsilateral and contralateral brain tissue (**d**) and additional selected stroke response genes. *n* = 4 mice per group. Scale bar indicates z-score normalized expression (**d**). Median ± interquartile range, ordinary one-way ANOVA with Tukey’s HSD test (**e**). (**f-k**) Transcriptional analysis of sdCAMs from WT stroke mice. Heatmap of all 2,346 DEGs (**f**) and volcano plots (**g**) of DEGs between ipsilateral and contralateral hemisphere, Benjamini–Hochberg adjusted *p* < 0.05 and |log2 fold change| > 0.59, NS: *p* > 0.05. Overlap of stroke response genes (**h**). Gene ontology (GO) terms derived from treatment-specific sdCAM stroke response genes (**i**) and selected stroke response genes (**j, k**). sdCAMs from two mice each were pooled; *n* = 3 (ispilateral), *n* = 4 (contralateral) biological replicates. Scale bar indicates z-score normalized expression (**f**). Median ± interquartile range and ordinary one-way ANOVA with Tukey’s HSD test (**j, k**). (**l-o**) Transcriptional analysis of microglia (MG) and sdCAMs from *Ccr2*^*CreERT2*^*R26*^*tdT*^ no stroke (∅) and stroke mice. Heatmap of all 642 DEGs between MG, tdT^−^ sdCAMs and tdT^+^ sdCAMs (**l**). GO terms derived from sdCAM subtype specific stroke response genes (**m**). DEGs in MG with Benjamini–Hochberg adjusted p values (**n**). Boxplots of selected genes (**o**). *n* = 3 (tdT^+^ sdCAMs BLZ945), *n* = 5 (all other groups). Scale bar indicates z-score normalized expression (**l**). Median ± interquartile range and ordinary one-way ANOVA with Tukey’s HSD test (**o**). Source data

Thus, we applied a model of thromboembolic stroke^47,48^ in WT mice after repopulation (Fig. 8a). Notably, repopulated mice performed worse at 1 day post stroke (+1 d) and +5 d in two independent neurological function paradigms compared to control animals (Fig. 8b, c). No differences in lesion volume, angiographic score, hemorrhage score, histological tissue examination or IBA1 immunoreactivity in the CNS parenchyma were apparent at +1 d (Extended Data Fig. 10a–c and Supplementary Fig. 6a–c). Moreover, the lesion sites were not infiltrated by neutrophils, T cells or B cells at +1 d (Supplementary Fig. 6d).

To explore the underlying mechanism of the neurological impairment, we investigated the transcriptional reaction to stroke at +1 d in whole CNS tissue. Immune response genes were strongly induced in the ipsilateral hemisphere, accounting for most upregulated DEGs (Extended Data Fig. 10d). We identified 115 upregulated DEGs in controls between ipsi- and contralateral hemispheres and 274 upregulated DEGs in repopulated mice (Fig. 8d, e). Of note, 266 of the 279 total upregulated DEGs showed higher mean expression in repopulated mice, including genes involved in macrophage activation, inflammation, reactive oxygen species (ROS) production and blood–brain barrier or extracellular matrix modification (Fig. 8f, g and Extended Data Fig. 10e).

Similar to the whole-tissue analysis, examination of transcriptional changes in sdCAMs at +1 d revealed a substantial induction of genes involved in immune responses (Extended Data Fig. 10f). Comparison to the contralateral hemispheres showed pronounced differences in the stroke response. We found 425 upregulated DEGs in sdCAMs from repopulated mice and 225 upregulated DEGs in control mice, of which 74 DEGs overlapped (Extended Data Fig. 10g, h). GO term analysis suggested a strong shift toward a proliferative and phagocytic phenotype in repopulated sdCAMs (Extended Data Fig. 10i). Gene-level analysis further supported the notion and suggested an increase in activation markers and ROS production (Extended Data Fig. 10j, k).

To disentangle treatment- and origin-dependent responses, we induced strokes in *Ccr2*^*CreERT2*^*R26*^*tdT*^ mice (Fig. 8h). Immunofluorescence revealed pronounced engraftment of HSC-derived tdT^+^ lmMΦ and pvMΦ, both in the cortical lesion as well as in the corresponding contralateral region in BLZ945-treated mice (Supplementary Fig. 6e). RNA-seq of tdT^+^ sdCAMs, tdT^−^ sdCAMs and microglia revealed upregulation of previously identified stroke response genes and downregulation of homeostatic markers (Extended Data Fig. 10l) compared to nonstroke-induced repopulated controls (Fig. 7a). Consistent with our previous results (Fig. 8f), repopulated sdCAM populations showed the highest expression of most stroke response genes, with tdT^+^ sdCAMs exceeding their tdT^−^ counterparts (Fig. 8i). The sdCAM populations from control and BLZ945-treated mice showed both common as well as subset-specific responses (Fig. 8j), with each gene set being associated with distinct GO terms (Extended Data Fig. 10m). Overlaying the stroke reactions of repopulated tdT^+^ and tdT^−^ sdCAMs revealed genes specifically regulated in tdT^+^ sdCAMs (Fig. 8k). In contrast, microglia showed no such discrepancy. DEG count in control microglia even slightly outnumbered that of repopulated microglia (Extended Data Fig. 10n). Supporting our previous analysis, we found increased expression of macrophage activation markers and chemokines, as well as *tdT* (*Gt(ROSA)26Sor*), in tdT^+^ sdCAMs (Fig. 8l and Extended Data Fig. 10o).

Finally, we performed molecular magnetic resonance imaging^42,47^ to determine whether the elevated cytokine and ROS production and macrophage activation translated into enhanced endothelial activation. While VCAM-1 signal intensity did not differ (Fig. 8m), the signal intensity of P-selectin, known to be upregulated under highly inflammatory conditions^42^, was significantly stronger in the ipsilateral hemisphere of repopulated mice and positively correlated with grip strength deficit (Fig. 8n, o).

Collectively, these results demonstrate that selective replacement of sdCAMs by HSC-derived macrophages influences immune response, cytokine production and functional outcome in a thrombin model of ischemic stroke.

## Discussion

In this study, we demonstrate that repopulated sdCAMs partially derive from transiently infiltrating but permanently engrafting HSC-derived CCR2^hi^Ly6C^hi^ monocytes. The resulting changes in the overall sdCAM transcriptome led to an altered reaction of sdCAMs to peripheral inflammatory stimuli and worsened the outcome following ischemic stroke.

Earlier studies had worked toward a method to efficiently and permanently replace CNS macrophages, microglia and sdCAMs, by HSC-derived cells. This was achieved through a combination of whole body irradiation (WBI) and bone-marrow transplantation (BMT) followed by CSF-1R-mediated depletion of CNS macrophages^24^. Other reports claimed to selectively replace sdCAMs with HSC-derived cells, at least temporarily, using WBI and BMT alone^26,27^; however, WBI has been shown to artificially prime the microglia niche for subsequent myeloid cell engraftment^23^. Deploying depletion and repopulation, we herein present a broadly applicable niche-specific permanent replacement strategy for directed and selective monocytic engraftment into CNS border niches.

While sdCAMs were strongly affected by their depletion and repopulation, we did not detect substantial changes in subdural or dural vascular niche cell types by scRNA-seq analysis. The predicted transient changes in the interactions between niche cells and macrophages, including increased CSF-1R signaling together with production of monocyte-attractant chemokines in repopulating macrophages, probably reflect the effort to restore homeostatic macrophage density within the CNS niches. These changes align with the infiltration of circulating monocytes. In accordance with known mechanisms of extravasation, the monocytic engraftment depended on integrin-mediated adhesion to ICAM-1 and VCAM-1, which were upregulated on vascular endothelial cells following macrophage depletion. Increased *Tnf* production by repopulating CNS macrophages might contribute to this vascular activation. Of note, microglia and sdCAMs remained separate during repopulation, with neither leaving its developmentally established niche.

Single-cell profiling revealed repopulated sdCAMs fall into the range of homeostatic YS-derived sdCAM subsets, but preferentially adopt an MHC class II^hi^ phenotype reminiscent of a previously described sdCAM state^5,49–51^. Bulk RNA-seq furthermore enabled us to define pan sdCAM markers (*Mrc1*, *Clec12a, Lyz2, Ms4a7* and *Apoe*) and unique signatures of HSC-derived (MHC class II-related genes, *Ccr2* and *Fxyd5)* and YS-derived (*Lyve1*, *Cd163* and *Folr2*) sdCAMs. This proposed set of markers allows the capture of sdCAM subtypes with implications for ontogeny. Chromatin accessibility was altered in all repopulated CNS macrophage populations; however, those alterations associated with proliferation or differentiation in response to depletion do not seem to be the main driver of the transcriptional changes seen in sdCAMs. Instead, ontogeny-related epigenetic imprinting remains the factor deciding the phenotype of sdCAMs.

While microglia exhibit a time-dependent transcriptional response to LPS^52,53^, sdCAM gene expression changes have only been studied in a *Lyve1*-expressing subset^54^. We therefore comprehensively analyzed overall and origin-specific responses of sdCAMs, dCAMs and microglia after repopulation. While many genes were commonly upregulated, we also identified cell-type-specific patterns. Notably, microglia and dCAMs remained unaffected by repopulation, whereas sdCAM responses were markedly altered. Experiments using *Ccr2*^−/−^ animals strongly suggested that these differences were mainly resulting from the engraftment of HSC-derived sdCAMs. It should be noted that due to the systemic route of LPS delivery, effects mediated by peripheral immune reactions cannot be fully ruled out as a confounding variable.

sdCAMs have been implicated in many CNS diseases, such as cerebral aneurysm formation^55^, ischemic stroke^45,47,51^ or neurodegenerative disorders^26,27,56,57^. Ischemic stroke induces a neuroinflammatory reaction that is thought to strongly contribute to the resulting neurological disabilities^58,59^. Accordingly, the increased expression levels of many cytokines, chemokines such as *Cxcl10*, and macrophage activation markers could explain the greater neurological impairment observed in repopulated mice. Notably, CXCL10 has been shown to directly impair synaptic plasticity via neuronal CXCR3, inducing sickness behavior in mice^60^. sdCAMs react rapidly to cerebral ischemia and switching their gene expression profile to an activated phenotype^46,51,61^. This immunological cascade can shape the outcome in either direction, worsening neurological deficits or dampening damage and aiding in resolution^47,62^. Age-related changes in sdCAM phenotype were recently shown to counteract increased ischemic stroke severity in aging mice^47^. Absence of sdCAMs has also been shown to increase stroke severity^45^. Our data further strengthen this theory by pointing to a protective role for YS-derived sdCAMs in stroke. Of note, CAMs are replaced long-lastingly by HSC-derived cells in models of traumatic brain injury and infection^30^. Consequently, we propose that such HSC-derived sdCAMs could be important players in case of a secondary insult.

In summary, the dual origin of replenished sdCAMs based on a single short-term depletion paradigm renders sdCAMs uniquely accessible for HSC-linked replacement strategies in CNS diseases.

## Methods

### Mice

All animal experiments were performed in compliance with respective national and federal regulations and approved by animal welfare authorities (Regierungspräsidium Freiburg, French Ministry of Higher Education and Research). C57BL/6JCrl (Charles River) served as WT mice. *Ccr2*-KO mice were from The Jackson Laboratory (B6.129S4-*Ccr2*^*tm1Ifc*^/J, Jax #004999). *Cx3cr1*^*CreERT2*^ (ref.^63^), *Cxcr4*^*CreERT2*^ (ref. ^39^), *Hexb*^*CreERT2*^ (ref. ^18^), *Mrc1*^*CreERT2*^ (ref. ^12^) and *Ccr2*^*CreERT2*^ (ref. ^24^) (Jax #035229) lines were crossed with *R26*^*tdT*^ (ref. ^64^) (Jax #007914) or *R26*^*YFP*^ (ref. ^65^) (Jax #006148) reporter lines. Mice were bred under specific-pathogen-free conditions and housed under a regular light–dark cycle at 20–22 °C and 50–60% humidity. Both sexes were included in each group. Age of first gavage was 10 ± 3 weeks.

### Tamoxifen treatment

TAM in corn oil (both Sigma-Aldrich) was injected i.p. using Cre-driver line-adjusted protocols: 1 mg TAM per 100 μl oil for 5 days (*Cxcr4*^*CreERT2*^*R26*^*tdT*^), 4 mg 200 μl^−1^ twice 48 h apart (*Cx3cr1*^*CreERT2*^*R26*^*tdT*^, *Mrc1*^*CreERT2*^*R26*^*tdT*^) in 5–9-week-old animals, 0.4 mg 20 µl^−1^ on P7 and P9 (*Hexb*^*CreERT2*^*R26*^*yfp*^) or 2 mg 200 μl^−1^ three times 48 h apart (*Ccr2*^*CreERT2*^*R26*^*tdT*^).

### BLZ treatment

BLZ945-HCl (Novartis) at 27.28 mg ml^−1^ in aqueous 20% hydroxypropyl-β-cyclodextrin (CTL; Sigma-Aldrich) solution was applied through oral gavage (200 mg kg^−1^ BW) daily for 7 days.

### Antibodies

Information about all antibodies used throughout the study can be found in Supplementary Table 1.

### Antibody injections

For adhesion blockade in vivo, antibodies or isotype controls were mixed in InVivoPure, pH 7.0 Dilution Buffer (BioXCell) injected i.p. (200 µg per antibody in 100 µl, every other day for seven injections) starting on the last day of gavage.

### Bone-marrow transplantations

Recipient mice were anesthetized with 20 µl of a 1:1 medetomidin (Domitor, Orion Pharma)/ketamine (Ketamin 10%, Serumwerk) mixture, positioned under a ~1-cm thick lead shield covering the head, and irradiated with 11 Gy (X-ray irradiator, Rad Source RS2000). After 24 h, femurs and tibias from sex- and age-matched donor mice (1:2 donor:recipient ratio) were flushed with ice-cold PBS, filtered (100 µm) and centrifuged (4 °C, 300*g*, 5 min). Red blood cells were lysed (1 ml eBioscience RBC lysis buffer, Thermo Fisher), washed and resuspended at 1 × 10⁵ cells per µl in PBS, filtered (100 µm), and ~15 × 10^6^ cells were injected intravenously into recipients. Mice were housed in IVC cages and received neomycin-sulfate drinking water (1.1 g l^−1^; Sigma-Aldrich) for 14 days. Blood chimerism was assessed 4 weeks post-transplantation.

### LPS injections

Mice were i.p. injected with LPS (Sigma-Aldrich, L2880) in PBS at 2.5 mg kg^−1^ bodyweight or PBS alone 12 h before killing.

### Thromboembolic stroke model

Purified mouse α-thrombin was injected into the middle-cerebral artery (MCA) to induce occlusion as previously described^48^. In brief, mice were anesthetized, fixed, craniotomized, the dura was removed and a glass micropipette was inserted into the MCA to inject 1 μl thrombin (2 IU μl^−1^ for WT). The pipette was withdrawn after 10 min for thrombus stabilization. Cerebral blood flow was monitored by laser Doppler up to 20 min after occlusion. Lesion volume was assessed by MRI at +1 d. Experiments followed STAIR recommendations. Group sizes were based on previous studies^47,48^. RNA-seq experiments were performed at two independent centers. Animals were excluded if they died perioperatively or had infarcts <5 mm³ (2 of 34 mice).

### MRI procedure

MRI was performed as previously described^48^. Mice were anesthetized and imaging was performed on a 7T Pharmascan scanner (Paravision v.6.0.1). T2-weighted multislice multiecho images (TE/TR 33/2,500 ms) were used to quantify lesion volumes with ImageJ. T2*-weighted sequences assessed hemorrhage (0–2 scale) and two-dimensional time-of-flight angiography (TE/TR 12/7 ms) evaluated MCA recanalization using a thrombolysis in cerebral infarction-like scoring system (0–3).

### Molecular magnetic resonance imaging

Molecular MRI was performed as previously described^42,47^. MPIOs were conjugated to anti-P-selectin or anti-VCAM-1 antibodies. Conjugation was verified by intrastriatal LPS injection followed by 3D T2*-weighted imaging. MRI was performed immediately after intravenous injection of conjugated MPIOs (200 μl, 2 mg Fe per kg) at 70-μm isotropic resolution (TE/TR 13.2/200 ms; flip angle 21°). Images were displayed as minimum intensity projections of six slices, and signal voids were quantified in ImageJ using automated thresholding and expressed as the percentage of void area within the region of interest.

### Corridor test

Sensorimotor performance was assessed using a corridor exploration task. A black PVC corridor (150 × 6 × 16 cm) with five objects (2 cm) on each side, spaced 18.5 cm apart, 1 cm above the floor was used. Object shape and color varied between test days. After 15 min habituation, mice explored the corridor freely for 1 min, and object explorations were recorded.

### Grip strength test

Forepaw grip strength was measured using a BIOSEB grip strength meter. Mice were placed at a T-bar and gently pulled by the tail. Measurements were taken at baseline (day before MCAO), and at 24 h and 5 days post-MCAO. Each session consisted of five trials with 1-min rest intervals, and mean force was calculated. Mean strength deficit relative to baseline was calculated for each mouse.

### Immunofluorescence

After transcardial perfusion with PBS, brains were fixed for 5–6 h in 4% PFA, put in 30% sucrose in PBS until saturation, embedded in Tissue-Tek O.C.T. (Sakura Finetek), frozen at −80 °C, sliced (Leica CM1900) at 20 µm (analysis) or 50 µm (representative images), rehydrated in PBS (5 min) and permeabilized for 1 h (5% bovine serum albumin and 0.5% Triton X-100 in PBS). Brain sections were processed free floating or on slides in upright racks. Primary antibodies were added (overnight, 4 °C) in blocking buffer. Secondary antibodies were applied (2 h). Coverslips were mounted with ProLong Diamond (Thermo Fisher Scientific) or with Mowiol.

### Immunohistochemistry and H&E staining

Chromogenic IHC^66^ and hematoxylin and eosin (H&E) staining were conducted on 3-µm-thick FFPE sections. IHC used the labeled streptavidin–biotin technique. The slides were deparaffinized in xylene, subjected to heat-induced antigen retrieval in EnVision low pH buffer (40 min) and treated with 3% hydrogen peroxide (Carl Roth, 8070.1) for 10 min. Blocking solution (10% normal goat serum (SouthernBiotec, 0060-01), 1% Triton X-100 (Sigma, T8787-100ML) and TRIS buffer (EnVision Flex Wash Buffer, DAKO, K8000), at pH 7.2, 1 h) was applied. The primary antibody was incubated overnight. Slides were washed and treated with secondary antibody (45 min) and washed. Streptavidin-HRP (7105-05, SouthernBiotech, 1:1,000 dilution in Tris buffer, 45 min) was applied. Sections were washed, exposed to DAB solution (one drop of EnVision Flex DAB Chromogen per 1 ml of EnVision Flex Substrate Buffer) and counterstained (Gill’s hematoxylin solution (Sigma, 1051750500). For H&E staining, slides were deparaffinized, rehydrated through graded ethanol to water and stained with Gill’s hematoxylin. After rinsing, sections were differentiated in 0.3% acid alcohol (1% HCl in 70% ethanol, 5–10 s), rinsed and blued (0.1% sodium bicarbonate, 1 min). Eosin Y (0.5% in ethanol) staining was performed (1 min), followed by dehydration through ascending ethanol and clearing in xylene (2 × 5 min). Coverslips were mounted with Vitro-Clud (R. Langenbrinck, 04-0001).

### Wholemounts

Leptomeninges were sliced off from fixed brains, cut into 3 × 3-mm pieces and processed as described under the immunofluorescence section.

### Microscopy

Imaging was conducted with a conventional fluorescence microscope (Keyence BZX or Leica Thunder Imager, quantification) or with a TCS SP8 X confocal (Leica, LAS X 3.5.7.23225, representative images) using a ×20 0.75 NA objective (HC PL APO 20x/0.75 IMM CORR CS2).

### Flow cytometry

Flow cytometry was conducted as described previously^3^. In brief, around 100 µl of blood were collected in PBS with 0,5 mol l^−1^ EDTA at pH 8.0 and lysed three times with Red Blood Cell Lysis Buffer (Thermo Fisher Scientific) before the staining procedure. Animals were then perfused transcardially with ice-cold PBS. MG and sdCAMs were isolated from whole brain for the experiments in Figs. 1 and 3, otherwise choroid plexuses were removed under a stereomicroscope. For digestion, brains were homogenized in HBSS containing 0.1 mg ml^−1^ DNase I (Roche) and 0.2 mg ml^−1^ Liberase (Roche) and put in a shaker at (37 °C, 1,000 rpm, 15 min). Cells were filtered, density centrifuged, cleaned of myelin, washed and used for the staining procedure. For dCAM data, cranial dura mater was removed from the cranial bone under a stereomicroscope and digested in 0.5 mg ml^−1^ Collagenase IV (Sigma-Aldrich) in PBS (30 min, 37 °C). Cell suspensions were mechanically dissociated, filtered, washed and used for the staining procedure. To analyze niche cells from different compartments, cranial dura, choroid plexus, leptomeninges and the cortical parenchyma were separated and enzymatically digested. Dura and the parenchyma were put into 0.2 mg ml^−1^ collagenase P (Sigma), 60 U ml^−1^ DNase I (Applichem), 0.3 U ml^−1^ Dispase I (Roche), 2% FCS and 20 mM HEPES in RPMI 1640 (all three Lonza) and digested (pH 7.2, 37 °C, 15 min, three cycles). For leptomeningeal dissociation, 0.2 mg ml^−1^ collagenase type 2, with two 5-min digestion steps was applied. Cells were then washed. From cortical samples, myelin was removed by density centrifugation. For the staining procedure, first, an Fc Block was applied (5 min, 4 °C) and samples were incubated with antibodies for 45 min (for blood 20 min, 4 °C). After washing, cells were sorted using an Aria III or analyzed using a LSRFortessa (Becton Dickinson). Dead cells were defined by labeling with Fixable Viability Dye (65-0866, eBioscience) or DAPI. Data were acquired with FACSDiva (Becton Dickinson). Post-acquisition analysis was performed using FlowJo v.10.10.

### Single-cell RNA-sequencing

After isolating whole brain and removing choroid plexuses under a stereomicroscope, for Fig. 2, cerebral cortex, leptomeninges and dural immune and niche cells were sorted by gating DAPI^−^ cells and then for CD11b^+^CD45^+^ (immune), CD31^+^ (endothelial), PDPN^+^ (fibroblasts) and marker-negative (other cell types) cells as described in the flow cytometry section and as seen in the gating strategies (Supplementary Fig. 1). For Fig. 5, only CD45^+^CD11b^+^Gr-1^−^CD11c^−^ cells were sorted from brain devoid of choroid plexus. Afterwards, the 10x scRNA-seq platform using the Chromium controller with the Chromium Next GEM Single Cell 3’ kit v.3.1 (Fig. 5) or Next GEM Single Cell 3’ HT kit v.3.1 (Fig. 2) (10x Genomics) was applied. Amplification of complementary DNA and library preparation were conducted following the manufacturer’s instructions. Libraries were sequenced on a NextSeq1000 Sequencer (Illumina) appropriate for obtaining 20,000 reads per cell. The resulting fastq files were further processed using the Cell Ranger v.7.1.0 pipeline (10x Genomics) for demultiplexing, read alignment to the mouse genome (GRCm39 or mm10; Cre, tdTomato, WPRE-QY added as chromosomes) and gene count determination.

### Doublet detection, quality control and analysis of the single-cell transcriptomic data

Transcriptomic data was analyzed in RStudio (build 764) with R programming language (v.4.4.0). The feature bc matrices were loaded with Seurat (v.5.0.3)^67^.

For Fig. 2, count matrices were filtered for low-quality cells (features 200–6,000, mitochondrial RNA < 5%) and doublets were removed using scDblFinder (v.1.16.0) after conversion with as.SingleCellExperiment^68^. Singlets were retained and merged into one Seurat object. Standard Seurat preprocessing was applied (NormalizeData, FindVariableFeatures (5,000), ScaleData, RunPCA, ElbowPlot, RunUMAP, FindNeighbors and FindClusters). Cells were manually inspected with CellSelector and cells expressing multiple lineage markers were removed^12,18,29^. Visualization used UMAP/DimPlot^69^. Batch correction was performed using Harmony^70^ (Split and IntegrateLayers), followed by the standard Seurat workflow using the Harmony reduction. The integrated object was converted to SingleCellExperiment and annotated with SingleR^71^. Major cell classes were subset (fibroblasts, endothelial, vascular smooth muscle cells, pericytes, immune and others) and subtypes manually annotated using FindAllMarkers with established literature^3,22,33–37^ and previous biological knowledge. CellChat^72^ was used to infer cell–cell interactions within the dura and leptomeninges/cortex. For macrophage-niche communication, CellChat objects were split into CTL, 5 d and 8w, top interaction partners were identified (excluding self-loops and niche–niche interactions) and interactions with homeostatic and repopulating macrophages were visualized.

For Fig. 5, processing was carried out as for Fig. 2, but cells were filtered for features 200–3,000 and mitochondrial RNA < 5% and Seurat preprocessing differed for FindVariableFeatures (2,000; excluding artificial, mitochondrial and ribosomal genes). A metadata column for tdTomato+WPRE-QY expression was added. Batch correction was performed using CCA^73^ (SplitObject, FindIntegrationAnchors and IntegrateData), followed by the standard Seurat workflow. Two additional low-quality clusters were removed based on low nCountRNA/nFeatureRNA and low gene expression. The standard Seurat workflow was applied to the updated object. Cell types were annotated based on known cell-type-specific marker genes, shown in Fig. 6a.

### CAM and MG subset analysis

CAMs and MG were subsetted, reintegrated by CCA, and processed using the standard Seurat workflow. The integrated data slot was used for downstream analysis. Clustering resolution was set to 0.25 for both. Heatmap genes were filtered for adjusted *P* < 0.05, log_2_fold change > 0.59, pct.1 > 0.2 and pct.1–pct.2 > 0.15, showing up to 30 genes per cluster. Volcano plots (BLZ945 versus CTL) were generated using FindMarkers (pct.min = 0.2). Cluster proportion changes were assessed using the propeller workflow (speckle)^74^. For CAMs, CAM2 was compared to CAM1 + 3 using FindMarkers (pct.min = 0.2). Overlapping DEGs (adj. *P* < 0.05, pct.1 > 0.2) between treatment and cluster comparisons were visualized using VennDiagram^75^; top ten genes up/down by log_2_fold change are shown. GO analysis was performed with EnrichGO (clusterProfiler) using significant cluster 2 versus 1 + 3 genes and nonzero genes as background, followed by simplify (cutoff of 0.7)^76^. YS-derived and HSC-derived gene modules were derived from bulk RNA-seq (adj. *P* < 0.05; log_2_fold change > 1 or <−1) and visualized in CAM subsets using AddModuleScore with FeaturePlot or DoHeatmap.

### Bulk RNA-seq of CNS macrophages and stroke tissue

CAMs, sdCAMs, dCAMs and MG were isolated by FACS from dissociated mouse brains (processed as described in the flow cytometry section) and sorted into RNA Protect Cell Reagent (QIAGEN); total RNA was extracted using the PicoPure RNA Isolation kit. First-strand cDNA was generated from 150–600 pg RNA using the SMARTer Ultra Low Input RNA kit v.4, followed by long-distance-PCR amplification (12–14 cycles), bead purification, Nextera XT library preparation, equimolar pooling and 50-cycle single-read sequencing on Illumina HiSeq 1000; reads were aligned to Gencode M36/mm10 or GRcm39 with STAR v.2.7.11a and counted with FeatureCounts v.2.0.8. In the post-repopulation experiment (Fig. 5), CAMs from *Mrc1*^*CreERT2*^*R26*^*tdT*^ mice were sorted (tdT⁺ and tdT⁻ CD206⁺). Lowly expressed genes (sum counts <10 in ≥75% of samples) were removed before performing DESeq2 differential expression, lfcShrink(apeglm), heatmaps of significant DEGs (*P*adj ≤ 0.05, |log_2_fold change | ≥ 1; *n* = 380), volcano plots and enrichGO analysis. For LPS stimulation, MG and (all or tdT⁺/tdT⁻) sdCAMs were isolated from *Ccr2*^*CreERT2*^*R26*^*tdT*^, WT or *Ccr2*^−/−^ mice 12 h after LPS (2.5 mg kg^−1^) or PBS. dCAMs were assessed in a separate experiment. Lowly expressed genes (sum count < sample number/2) were filtered, ComBat-seq batch correction was applied to WT/*Ccr2*^−/−^ datasets (batch = batch, group = treatment; covariates = genotype, cell type). To ensure the quality of RNA-seq analysis and focus on immune response-related gene expression, genes in the KEGG_ribosome pathway, KEGG_oxidative_phosphorylation pathway (msigdbr), biomaRt pseudogenes, predicted genes, novel or uncharacterized genes as well as biomaRt noncoding genes (lincRNA, miRNA, snRNA and snoRNA) and genes with substrings associated with such pathways (‘Mrp’, ‘Rpl’, ‘Rps’, ‘Sn’, ‘Sf’, ‘Gtf’, ‘Med’, ‘Rbm’, ‘Rik’, ‘Atp’, ‘Nduf’, ‘Tomm’, ‘Pol’, ‘Anap’, ‘Rna’, ‘Dna’, ‘Gm’, ‘Ddx’, ‘Smim’, ‘Bola’, ‘Timm’ and ‘Micos’) were removed. DESeq2 was used for differential expression (MG excluded for CAM-specific analyses), with PCA and heatmaps on variance-stabilized top variable genes, apeglm-shrunken volcano plots, enrichGO on DEGs (*P*adj ≤ 0.05) and ggradar plots of selected LPS-responsive genes. In the stroke experiment (Fig. 8), at 24 h post-MCAO, brains from WT or *Ccr2*^*CreERT2*^*R26*^*tdT*^ mice were sectioned, choroid plexus removed, and CAMs sorted as Ly6C⁻Ly6G⁻CD11c⁻CD45⁺CD11b⁺CD206⁺ (tdT⁺/tdT⁻ in reporter mice) or MG as CD206⁻ counterparts (cells of two BLZ945 mice were pooled per replicate); whole-tissue RNA was extracted using RNeasy Micro kit with DNase and Bioanalyzer quality control. Lowly detected genes (sum count < sample number/2) were filtered, DESeq2 performed differential expression, PCA, apeglm-based volcano plots and GO analysis on upregulated genes (*P*adj ≤ 0.05) in ipsilateral hemisphere (CTL or BLZ945); additional visualizations included Venn diagrams of overlapping upregulated genes, bar plots of gene counts with higher stroke-induced expression, box plots of selected immune genes. Volcano plots for *Ccr2*^*CreERT2*^*R26*^*tdT*^ data were generated by plotting DEGs detected in repopulated stroke MG versus CTL MG and overlaying DEGs detected in vehicle stroke MG versus CTL MG or by plotting DEGs detected in tdT^+^ stroke CAMs versus tdT^+^ CTL CAMs and overlaying DEGs detected in tdT^−^ stroke CAMs versus tdT^−^ CTL CAMs.

### scATAC-seq

MG, sdCAMs, dCAMs and Ly6C^hi^ monocytes were isolated from brains or blood of *Mrc1*^*CreERT2*^*R26*^*tdT*^ mice 8 weeks after depletion as described under flow cytometry. Then, 3–4 biological replicates were pooled, MG and monocytes from the same condition were pooled. Cells were sorted and processed using the Chromium Single Cell ATAC-seq platform (10x Genomics). Nuclei were isolated following the 10x Genomics protocol (CG000366, Rev A) with minor adaptations. In brief, sorted cells were lysed in diluted lysis buffer, quenched with wash buffer, centrifuged, resuspended and filtered (10-µm strainer). Nuclei integrity was assessed by DAPI staining and counted using Trypan Blue on a Countess automated cell counter. Encapsulation and library preparation were performed using Chromium Next GEM Single Cell ATAC Reagent Kits v.2 (10x Genomics). Library quality was assessed by Qubit and Bioanalyzer. Raw reads were processed using Cell Ranger ATAC (v.2.2.0) with alignment to the mm10 genome and generation of single-cell accessibility matrices. Downstream analysis was performed in R using Signac (v.1.16). Feature-barcode matrices were loaded, and quality control was performed based on transcription start site enrichment and nCount_peaks. Samples were merged based on a common GRanges peak set. Standard Signac workflow (RunTFIDF, FindTopFeatures, RunSVD, RunUMAP, FindNeighbors and FindClusters) was applied. Gene activity scores were calculated. Cell type annotation was transferred from scRNA-seq data (Fig. 2; macrophages and monocytes from CTL and 8w samples) using TransferData and refined using predicted identities and sorting metadata. Peak calling was performed using CallPeaks (MACS2; group.by = cell type, combine.peaks = TRUE), followed by removal of nonstandard chromosomes, quantification (FeatureMatrix) and annotation (RegionStats). A standard Signac workflow and gene activity calculation were repeated on peak assay. The bar graph of differential accessibility includes increased and decreased peaks. Module contents were based on (sc)RNA-seq analysis and FindAllMarkers results. Motif enrichment was performed using JASPAR2020, and transcription factor activity was assessed using chromVAR. Visualizations were generated with SCpubR^77^, Signac, EnhancedVolcano^78^ and ggplot2 (ref. ^79^).

### Analysis and experimental information

Analysis, surgeries and outcome assessments were conducted blinded to treatment conditions. Animals were randomly assigned to treatment groups, with a similar number of males and females per group. Sample sizes were not predetermined, but are similar to those reported previously^3,12,47^.

### Histological analysis

All data points for histological analyses in Figs. 1 and 3 and associated Extended Data derive from manual quantification (ImageJ Fiji and Cell Counter plug-in) of ten regions of interest for cerebral cortex, at least six for cerebellum and at least four for olfactory tubercle per biological replicate. In brief, the laminin channel was activated and parenchyma area measurements and leptomeningeal length measurements were performed, the IBA1 channel was used to count the number of MG. Next, the CD206 channel was used to quantify the number of lmMΦ and pvMΦ. Last, the reporter channel was activated and tdT^+^ cells were quantified. For Fig. 4, images of whole-brain sagittal sections were semi-automatically analyzed using QuPath v.4.4 software (≥1,000 CAMs per biological replicate). For Fig. 5, whole-brain sagittal sections were manually counted using ImageJ Fiji (≥1,000 CAMs per biological replicate). For analysis of CD62P^+^, CD62E^+^ or ICAM-1^+^ vessels, Cell Profiler v.4.2.5 (Crop, Threshold, IdentifyPrimaryObjects, MaskObjects and MeasureImageAreaOccupied) was deployed.

### Statistical analysis

Normality and homoscedasticity were generally assumed; equality of variances was assessed for all GraphPad-based analyses. A significance level of 5% was used (*P* < 0.05). Histological and flow cytometry data were analyzed in GraphPad Prism v.11 using unpaired two-tailed *t*-tests or one-way ANOVA with Šidák’s or Tukey’s post hoc tests. For Fig. 1f, cell density data were analyzed in R using biological replicate-level counts. Data were converted to long format and Welch’s two-sample *t*-tests were performed for each day (CTL versus BLZ), followed by Bonferroni correction across six comparisons. A three-parameter logistic growth model was fitted to mean depletion over time using nonlinear least squares:

$$\mathrm{Depletion}( \% )=L/(1+\exp (-k\times (\mathrm{day}-{t}_{0}))),$$

where *L* is the asymptote, *k* is the growth rate and *t*₀ is the inflection point. Convergence failures were handled with tryCatch. Model fit was evaluated (*R*²) and time to 90% depletion (*t*₉₀) was derived analytically. Sequencing data were analyzed in R using methods described in the respective RNA-seq sections. Cluster composition in Figs. 2 and 5 was assessed with the propeller workflow (speckle), applying arcsine square-root transformation followed by a *t*-test (Fig. 5e, two conditions) or ANOVA (Extended Data Fig. 1c; three conditions) with Benjamini–Hochberg FDR correction. Corridor test discrete number statistics were performed with gstat and significance was tested with the Brunner–Munzel test. P-selectin MPIO signal and strength deficit correlation was evaluated using Spearman correlation and linear regression (lawstat). Box plots were analyzed by ANOVA with Tukey’s test. Discrete number graphs were analyzed by chi-squared testing.

### Reporting summary

Further information on research design is available in the Nature Portfolio Reporting Summary linked to this article.

## Online content

Any methods, additional references, Nature Portfolio reporting summaries, source data, extended data, supplementary information, acknowledgements, peer review information; details of author contributions and competing interests; and statements of data and code availability are available at 10.1038/s41590-026-02457-y.

## Supplementary information

Supplementary Information Supplementary Figs. 1–6 and Table 1

Reporting Summary

Supplementary Source Data Source Data for Supplementary Figures.

## Source data

Source Data Fig. 1 Statistical source data for Fig. 1.

Source Data Fig. 3 Statistical source data for Fig. 3.

Source Data Fig. 4 Statistical source data for Fig. 4.

Source Data Fig. 5 Statistical source data for Fig. 5.

Source Data Fig. 8 Statistical source data for Fig. 8.

Source Data Extended Data Fig. 1 Statistical source data for Extended Data Fig. 1.

Source Data Extended Data Fig. 2 Statistical source data for Extended Data Fig. 2.

Source Data Extended Data Fig. 3 Statistical source data for Extended Data Fig. 3.

Source Data Extended Data Fig. 4 Statistical source data for Extended Data Fig. 4.

Source Data Extended Data Fig. 10 Statistical source data for Extended Data Fig. 10.

## Data Availability

The (sc)RNA-seq and scATAC-seq data that support the findings of this study were deposited in the Gene Expression Omnibus under accession numbers GSE318650, GSE295335, GSE294773, GSE318386, GSE318071, GSE318159, GSE294912 and GSE295334. Source data are provided with this paper.

## Code availability

Code for statistical analyses is available at https://github.com/fliegaufm/Repopulation_of_CAMs.git.
