# Supplementary material for: Distinct origins and niches determine the cellular responsiveness of CNS macrophages after repopulation
Source: Nat Immunol. 2026 Mar 18;27(5):961–74. doi: 10.1038/s41590-026-02457-y (PMC13132723; doi:10.1038/s41590-026-02457-y)
Supplement: Supplementary file 1 — Supplementary Figs. 1–6 and Table 1 [file 41590_2026_2457_MOESM1_ESM.pdf]

nature immunology

Supplementary information

https://doi.org/10.1038/s41590-026-02457-y

Distinct origins and niches determine the cellular responsiveness of CNS macrophages after repopulation

In the format provided by the authors and unedited

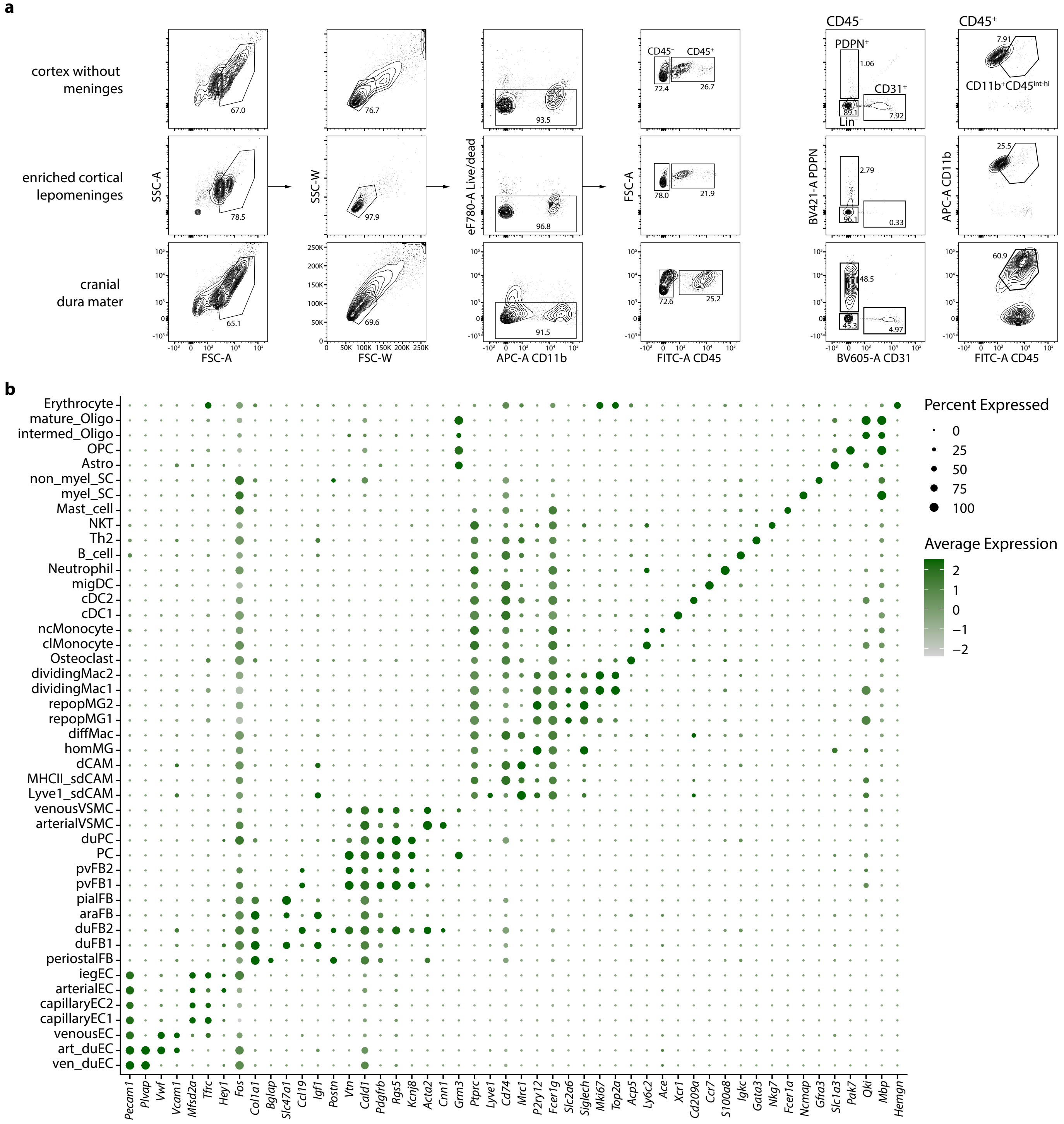

Supplementary Fig. 1 | Gating strategy and cluster markers of myeloid cells and niche cells. a, Representative gating strategies to sort CD31⁺, PDPN⁺, CD11b⁺CD45^{int-hi} and Lin⁻ cells for single-cell RNA-sequencing from the cranial dura mater, enriched cortical leptomeninges and cortex without leptomeninges of control C57Bl/6 mice or animals 5 days (5d) and 8 weeks (8w) after treatment with BLZ945. **b**, Dot plot depicting selected cell type defining markers. Color scale: Relative expression levels.

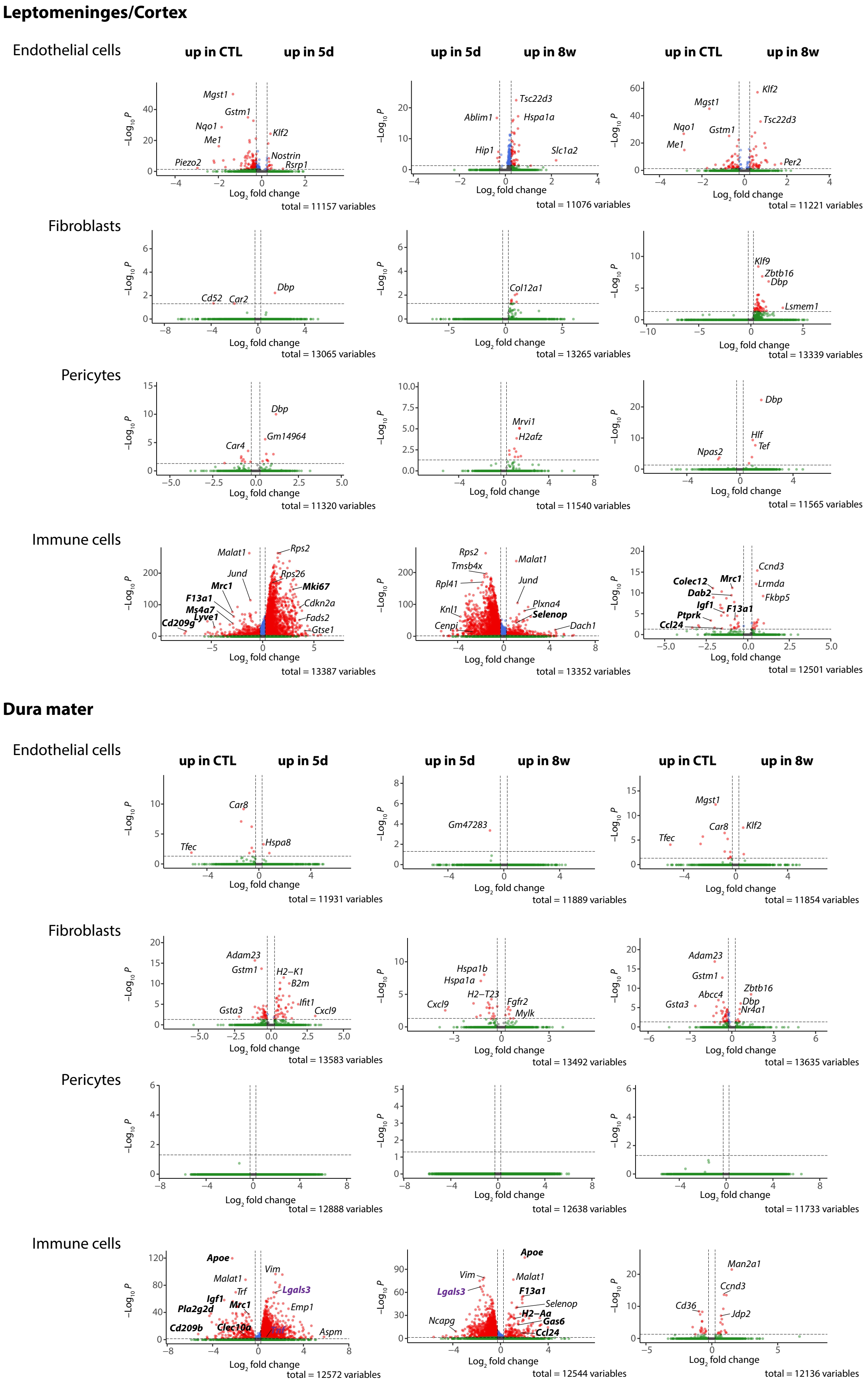

Supplementary Fig. 2 | Transcriptional alterations in CAM niches throughout repopulation. Volcano plots depicting differentially expressed genes in grouped niche cell types (endothelial cells, fibroblasts and pericytes) and immune cells between CTL and 5d and 8w post BLZ945 subdurally (top) and inside the dura mater (bottom). Genes in bold represent CAM marker genes, genes in violet represent cell division and macrophage activation markers.

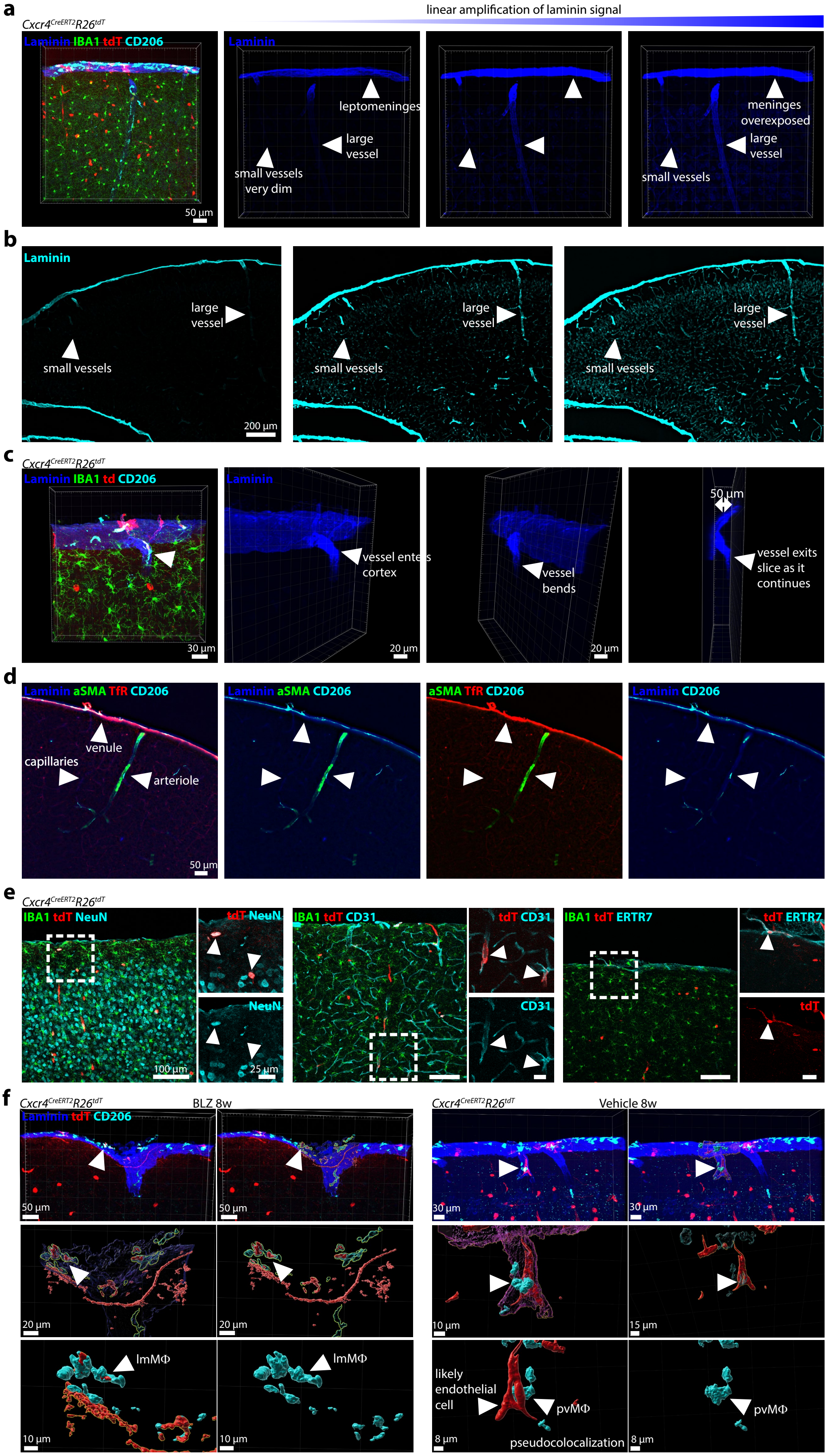

Supplementary Fig. 3 | Immunofluorescence imaging of sdCAMs and brains of *Cxcr4*^{CreERT2}*R26*^{tdT} mice. a, Representative Laminin immunofluorescence in ultraviolet channel (405 nm) exhibiting increased revelation of cortical small vasculature, neurons and overexposure of leptomeninges with linear amplification of signal. **b**, Representative Laminin immunofluorescence in infrared channel (647 nm) exhibiting increased revelation of cortical small vasculature, neurons and overexposure of leptomeninges with linear amplification of signal. **c**, Representative Laminin immunofluorescence in 50 µm cortical slice showing penetrating leptomeningeal vasculature that runs diagonally through the slice. **d**, Representative immunofluorescence of Laminin, endothelial subtype markers for arteries (a-smooth muscle actin) and veins (transferrin-receptor) and CD206. Weakly visible is the arteriole branching off into cerebral small vasculature. Arteriole covered with pvMΦ which are absent in small vasculature. **e**, Representative immunofluorescence of recombined cell types in the *Cxcr4*^{CreERT2}*R26*^{tdT} mouse line. Reporter labeling can be identified in NeuN^{+} neurons, CD31^{+} endothelial cells and ERTR-7^{+} fibroblasts. Arrows point to tdT^{+} cells also expressing the respective non-myeloid marker. **f**, 3D reconstructions of representative immunofluorescence images depicted in **Fig. 3g**. True colocalization of tdT and CD206 can be found 8w post BLZ945 treatment, while merely pseudo-colocalization can be found in Vehicle treated animals due to high background recombination in the *Cxcr4*^{CreERT2}*R26*^{tdT} mouse line. Arrows point to colocalization and pseudo-colocalization respectively.

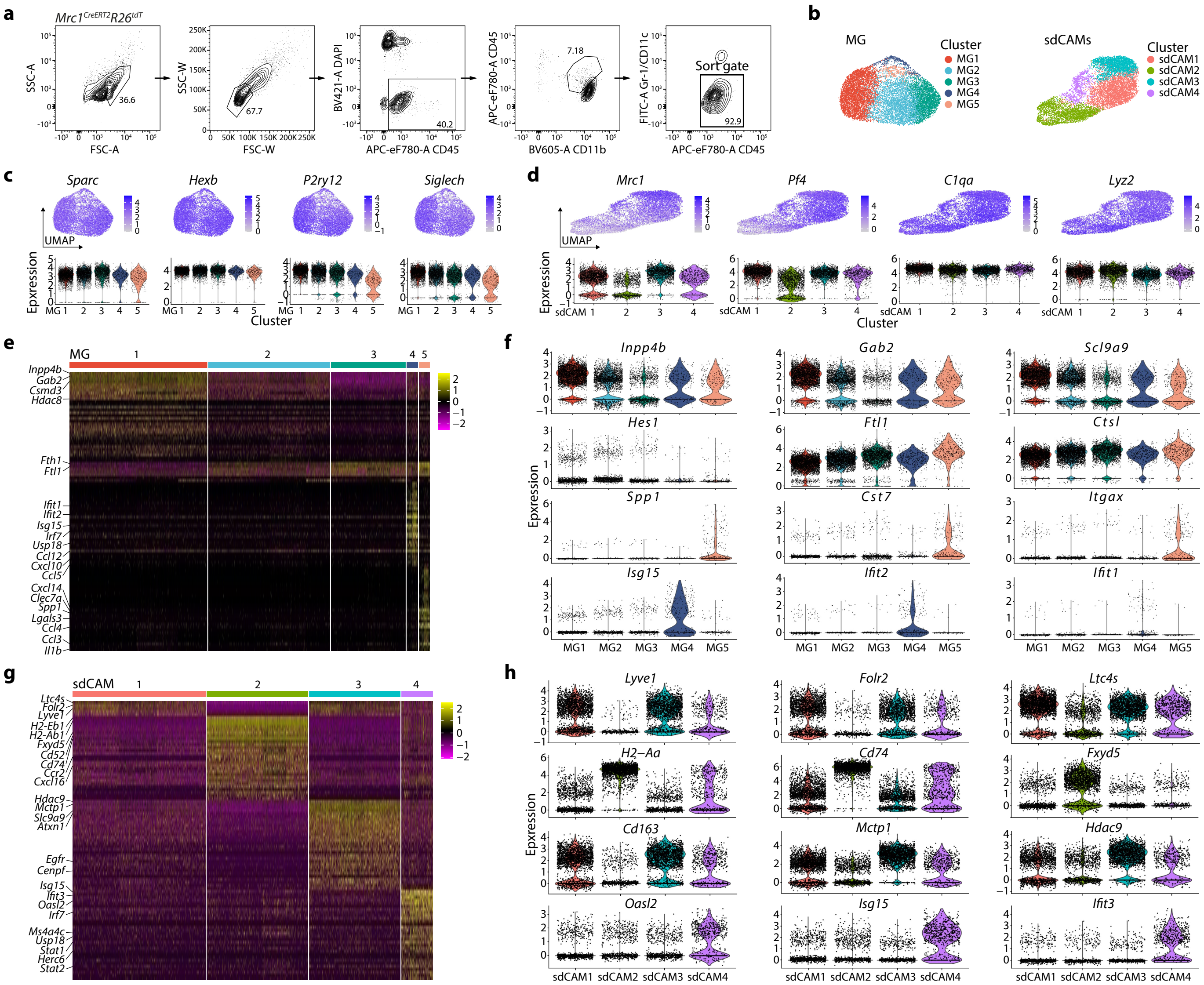

Supplementary Fig. 4 | Cluster markers of MG and sdCAMs. a, Representative gating strategy to sort Lin⁻CD11b⁺CD45⁺ cells for single-cell RNA-sequencing from the brains of control *Mrc1*^{CreERT2}*R26*^{tdT} mice or animals 8 weeks after treatment with BLZ945. **b**, Reclustered UMAPs of microglia (MG) and CAMs with cluster identities. **c**, **d**, Feature plots and violin plots depicting the expression of various cell type and macrophage marker genes in MG (**c**) and CAMs (**d**) within the distinct clusters. Scale bars show z-scores of minimum to maximum scaled gene expression. **e**, **f**, Heatmaps of the maximally top 30 significant differentially expressed genes (DEGs; Bonferroni adjusted *p*-value < 0.05) per cluster between the MG clusters 1-5 and violin plots of selected DEGs. Scale bar shows z-scores of minimum to maximum scaled gene expression. **g**, **h**, Heatmaps of the maximally top 30 significant DEGs (Bonferroni adjusted *p*-value < 0.05) per cluster between the CAMs clusters 1-4 and violin plots of selected DEGs. Scale bar shows z-scores of minimum to maximum scaled gene expression.

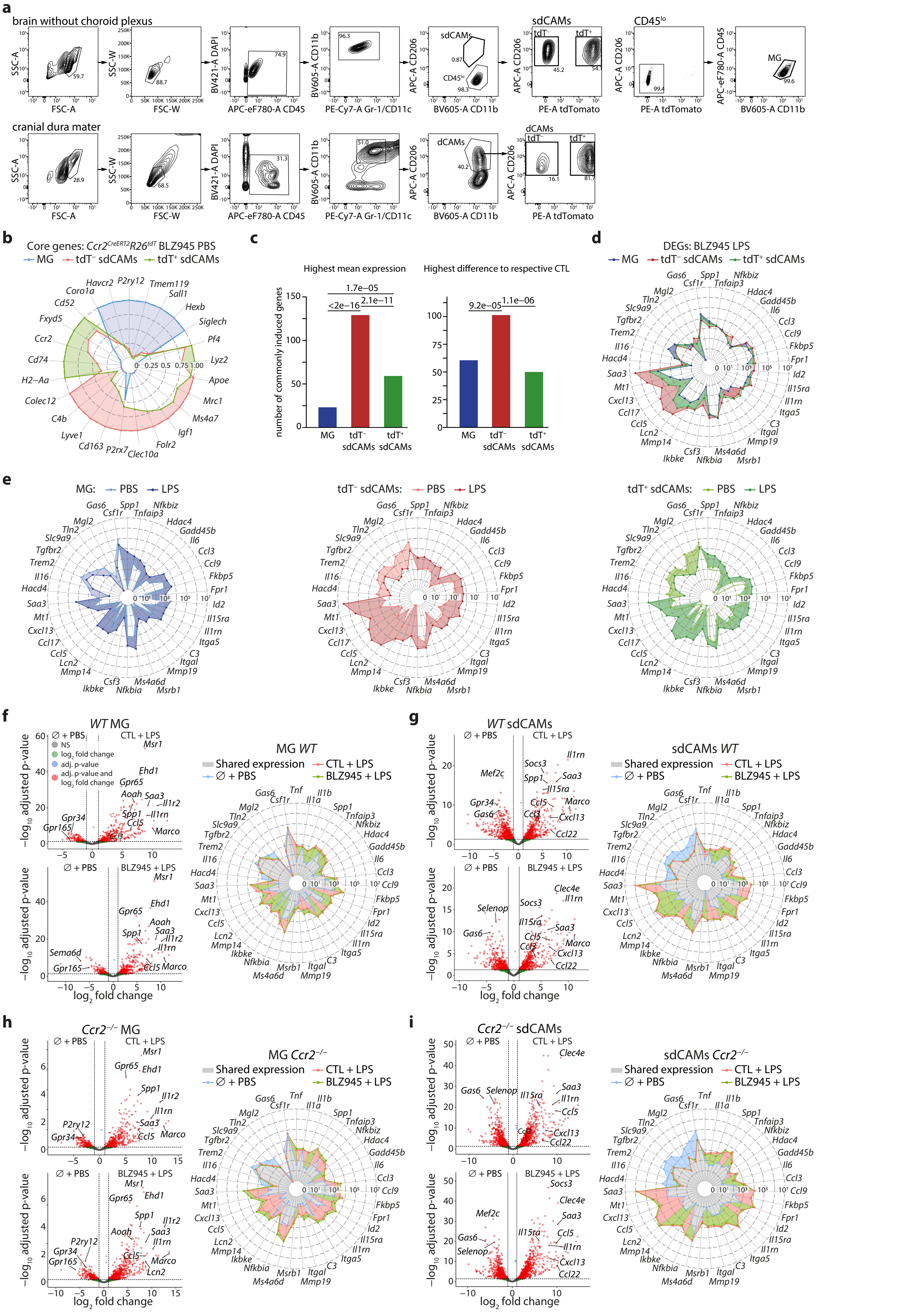

Supplementary Fig. 5 | Divergent reaction of distinct brain macrophage populations to LPS challenge. a, Representative gating strategy to sort microglia (MG), tdT$^-$ subdural (sd)CAMs and tdT$^+$ sdCAMs as well as tdT$^-$ dura (d)CAMs and tdT$^+$ dCAMs for bulk RNA-sequencing from the brains of control or LPS induced C57BL/6, *Ccr2*$^{-/-}$ or *Ccr2*^{CreERT2}*R26*^{tdT} mice. **b**, Linear scaled radar chart of selected core genes in BLZ945-treated *Ccr2*^{CreERT2}*R26*^{tdT} mice. **c**, Number of commonly upregulated LPS-induced genes with either highest mean expression or highest difference to respective control. Chi-square goodness-of-fit test with Benjamini-Hochberg adjusted p-values. **d**,**e**, Logarithmically scaled radar charts depicting normalized expression levels of selected genes following systemic LPS challenge (**d**) and for control (CTL) versus LPS challenge (**e**). **f**,**g**, Left: Differentially expressed genes (DEGs, Benjamini-Hochberg adjusted p-values, NS: $p > 0.05$) between MG (**f**) or sdCAMs (**g**) following systemic LPS challenge compared to untreated (∅) PBS-injected mice. Right: Logarithmically scaled radar charts depicting normalized expression levels of selected genes. **h**,**i**, Left: DEGs (Benjamini-Hochberg adjusted p-values, NS: $p > 0.05$) between MG (**h**) or sdCAMs (**i**) following systemic LPS challenge compared to ∅ PBS-injected mice. Right: Logarithmically scaled radar charts depicting normalized expression levels of selected genes.

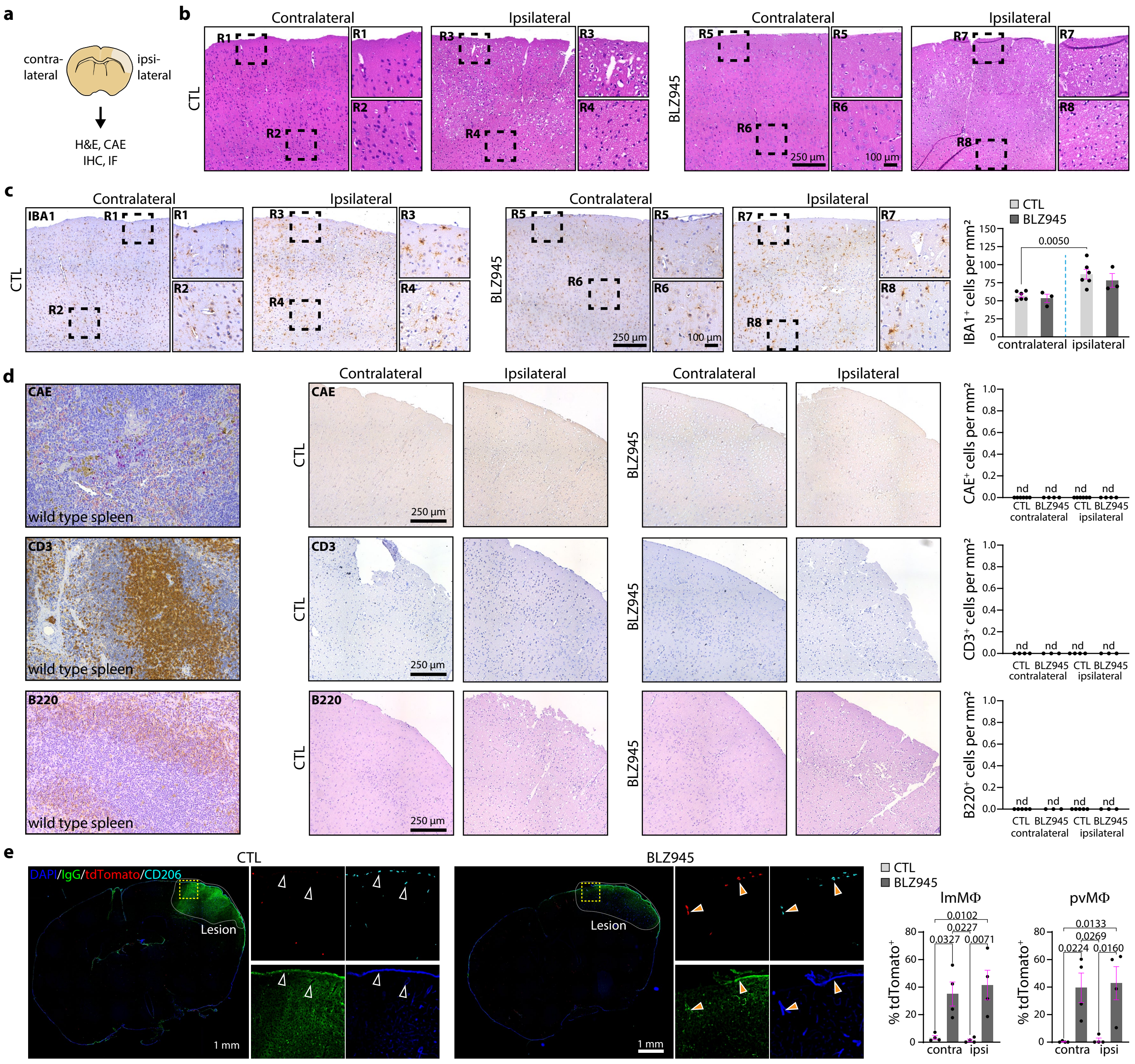

Supplementary Fig. 6 | Absence of neuroinflammation 1 day after stroke. a, Brain slices from animals subjected to stroke were either hematoxylin and eosin (H&E) or chloracetate esterase (CAE) stained or used for immunohistochemistry (IHC). **b**, Representative images of H&E staining of ipsilateral and contralateral hemispheres at day 1 (+1d) after stroke. **c**, Representative immunohistochemistry for IBA1 and quantification of parenchymal IBA1$^+$ cells. Symbols represent individual mice, *n* = 6 (CTL), *n* = 3 (BLZ945), mean ± SEM. Ordinary one-way ANOVA with Šidák-adjusted multiple-comparisons *p*-values. **d**, Representative CAE staining (top) and immunohistochemistry for CD3 (middle) and B220 (bottom) and quantification of positive cells in the parenchyma. Symbols represent individual mice, *n* = 6 (CTL CAE), *n* = 5 (CTL B220), *n* = 4 (BLZ945 CAE, CD3 CTL), *n* = 3 (CD3 BLZ945, B220 BLZ945). nd = not detected. Spleen of wild type mice served as positive controls (left panel). **e**, Representative images showing tdT+ leptomeningeal (lmMΦ) or perivascular pvMΦ) macrophages in coronal brain sections from control (CTL) or BLZ945 treated *$Ccr2^{CreERT2}R26^{tdT}$* mice at day 1 after thromboembolic stroke (related to **Fig. 8h-l**). Quantification of tdT$^+$ lmMΦ and tdT$^+$ pvMΦ in contralateral and ipsilateral hemispheres. Arrowheads: orange = tdT$^-$ lmMΦ and tdT$^-$ pvMΦ, open = tdT$^+$ lmMΦ and tdT$^+$ pvMΦ. Symbols represent individual mice, *n* = 4 per group, mean ± SEM. Ordinary one-way ANOVA with Šidák-adjusted multiple-comparisons *p*-values.

Section	Target / Antigen	Clone	Catalog #	Supplier / Source	Dilution	Notes / Application Context	Validation info
Antibody injections (in vivo blockade)	VCAM-1 (CD106)	M/K-2.7	BE0027	BioXCell	2 µg / µl	In vivo adhesion blockade; 200 µg in 100 µl i.p., every other day ×7 injections	https://bioxcell.com/invivomab-anti-mouse-cd106-vcam-1-be0027
	ICAM-1 (CD54)	YN1/1.7.4	BE0020-1	BioXCell	2 µg / µl	In vivo adhesion blockade; same dosing as above	https://bioxcell.com/invivomab-anti-mouse-cd54-icam-1-be0020-1
	VLA-4 (CD49d)	PS/2	BE0071	BioXCell	2 µg / µl	In vivo adhesion blockade; same dosing as above	https://bioxcell.com/invivomab-anti-mouse-human-vla-4-cd49d-be0071
	HRP	HRPN	BE0088	BioXCell	200 µg in 100 µl i.p., every other day ×7 injections (matched to blocking Abs)	Isotype control (for rat IgG1)	https://bioxcell.com/invivomab-rat-igg1-isotype-control-anti-horseradish-peroxidase-be0088
	KLH	LTF-2	BE0090	BioXCell	200 µg in 100 µl i.p., every other day ×7 injections (matched to blocking Abs)	Isotype control (for rat IgG2b)	https://bioxcell.com/invivomab-rat-igg2b-isotype-control-anti-keyhole-limpet-hemocyanin-be0090
Molecular Magnetic Resonance Imaging (mMRI)	P-selectin (for MPIO conjugation)	polyclonal	AF737	R&D Systems	N/A	Conjugated to MPIOs for in vivo imaging; no direct IF/IHC use here	https://www.rndsystems.com/products/mouse-rat-p-selectin-cd62p-antibody_af737
	VCAM-1 (for MPIO conjugation)	clone 429	553330	BD Biosciences	N/A	Conjugated to MPIOs for in vivo imaging	https://www.bdbiosciences.com/en-us/products/reagents/flow-cytometry-reagents/research-reagents/single-color-antibodies-ruo/purified-rat-anti-mouse-cd106.553330?tab=product_details
Immunofluorescence (IF) primary	Laminin	polyclonal	L9393	Merck Millipore	1:1000	Overnight at 4°C in blocking buffer	https://www.merckmillipore.com/ER/en/product/sigma/l9393
	Collagen IV	polyclonal	AB769	Millipore	1:500	Overnight at 4°C	https://www.merckmillipore.com/DE/de/product/Anti-Collagen-Type-IV-Antibody,MM_NF-AB769
	IBA1	Gp311H9	234 308	Synaptic Systems	1:500	Overnight at 4°C	https://sysy.com/product/234308
	CD206	MR5D3	MCA2235	Biorad	1:500	Overnight at 4°C	https://www.bio-rad-antibodies.com/monoclonal/mouse-cd206-antibody-mr5d3-mca2235.html?f=purified
	CD31	polyclonal	AF3628	R&D Systems	1:200	Overnight at 4°C	https://www.rndsystems.com/products/human-mouse-rat-cd31-pecam-1-antibody_af3628
	ICAM-1 (CD54)	G-5	sc-8439	Santa Cruz	1:200	Overnight at 4°C	https://www.scbt.com/de/p/icam-1-antibody-g-5?srsltid=AfmBOorNHA-sdBrxI66VDsxmzWbOr7j-nlX6DJK30EoJj6ZYM6QkFZhf
	P-selectin (CD62P)	polyclonal	AF737	R&D Systems	1:200	Overnight at 4°C	https://www.rndsystems.com/products/mouse-rat-p-selectin-cd62p-antibody_af737
	GFP	polyclonal	ab13970	Abcam	1:1000	Overnight at 4°C	https://www.abcam.com/en-us/products/primary-antibodies/gfp-antibody-ab13970
	Ki-67	polyclonal	ab15580	Abcam	1:250	Overnight at 4°C	https://www.abcam.com/en-us/products/primary-antibodies/ki67-antibody-ab15580
	MHC II	M5/114.15.2	107622	BioLegend	1:200	Overnight at 4°C	https://www.biolegend.com/en-us/products/alexa-fluor-700-anti-mouse-i-a-i-e-antibody-3413
Immunofluorescence (IF) secondary	mouse IgG	polyclonal	A10037	Thermo Fisher	1:500	2 hours at 25° C	https://www.thermofisher.com/antibody/product/Donkey-anti-Mouse-IgG-H-L-Highly-Cross-Adsorbed-Secondary-Antibody-Polyclonal/A10037
	rabbit IgG	polyclonal	ab175651	abcam	1:500	2 hours at 25° C	https://www.abcam.com/en-us/products/secondary-antibodies/donkey-rabbit-igg-h-l-alexa-fluor-405-ab175651
	guinea pig IgG	polyclonal	A-11073	Molecular Probes	1:500	2 hours at 25° C	https://www.thermofisher.com/antibody/product/Goat-anti-Guinea-Pig-IgG-H-L-Highly-Cross-Adsorbed-Secondary-Antibody-Polyclonal/A-11073
	rat IgG	polyclonal	SA5-10029	Thermo Fischer	1:500	2 hours at 25° C	https://www.thermofisher.com/antibody/product/Donkey-anti-Rat-IgG-H-L-Cross-Adsorbed-Secondary-Antibody-Polyclonal/SA5-10029
	goat IgG	polyclonal	A11057	ThermoFisher	1:500	2 hours at 25° C	https://www.thermofisher.com/antibody/product/Donkey-anti-Goat-IgG-H-L-Cross-Adsorbed-Secondary-Antibody-Polyclonal/A-11057
Immunohistochemistry (IHC, chromogenic on FFPE)	IBA-1	EPR16588	178846	Abcam	1:1000	Overnight; LSAB technique	https://www.abcam.com/en-us/products/primary-antibodies/iba1-antibody-epr16588-microglia-marker-ab178846
	CD3	CD3-12	MCA1477	Biorad	1:100	Overnight; LSAB technique	https://www.bio-rad-antibodies.com/monoclonal/human-cd3-antibody-cd3-12-mca1477.html?f=purified
	B220 (CD45R)	RA3-6B2	557390	BD Biosciences	1:200	Overnight; LSAB technique	https://www.bdbiosciences.com/en-nl/products/reagents/flow-cytometry-reagents/research-reagents/single-color-antibodies-ruo/purified-rat-anti-mouse-cd45r.557390?tab=product_details
	rabbit IgG	polyclonal	4058-08	SouthernBiotech	1:300	45 min at 25° C	https://www.southernbiotech.com/goat-anti-rabbit-igg-fab-mouse-human-sp-ads-biot-4058-08
Flow Cytometry	Fc Block (CD16/32)	2.4G2	553141	BD Biosciences	1:25	5 min, 4°C	https://www.bdbiosciences.com/en-eu/products/reagents/western-blotting-and-molecular-reagents/purified-rat-anti-mouse-cd16-cd32-mouse-bd-fc-block.553141?tab=product_details
	CD11b (Integrin αM)	M1/70	101237	BioLegend	1:300	45 min, 4°C; 20 min for blood	https://www.biolegend.com/en-us/products/brilliant-violet-605-anti-mouse-human-cd11b-antibody-7637
	CD45	30-F11	561037	BD Biosciences	1:200	45 min, 4°C; 20 min for blood	https://www.bdbiosciences.com/en-pl/products/reagents/flow-cytometry-reagents/research-reagents/single-color-antibodies-ruo/apc-cy-7-rat-anti-mouse-cd45.561037?tab=product_details
	Ly6C	AL-21	561237	BD Biosciences	1:200	45 min, 4°C; 20 min for blood	https://www.bdbiosciences.com/en-pt/products/reagents/flow-cytometry-reagents/research-reagents/single-color-antibodies-ruo/alexa-fluor-700-rat-anti-mouse-ly-6c.561237?tab=product_details
	Ly6G	1A8	565964	BD Biosciences	1:300	45 min, 4°C; 20 min for blood	https://www.bdbiosciences.com/en-de/products/reagents/flow-cytometry-reagents/research-reagents/single-color-antibodies-ruo/buv395-rat-anti-mouse-ly-6g.565964?tab=product_details
	Gr-1 (Ly6G/Ly6C)	RB6-8C5	563849	BD Biosciences	1:300	45 min, 4°C; 20 min for blood	https://www.bdbiosciences.com/en-fi/products/reagents/flow-cytometry-reagents/research-reagents/single-color-antibodies-ruo/buv395-rat-anti-mouse-ly-6g-and-ly-6c.563849?tab=product_details
	CD115 (CSF1R / M-CSFR)	AFS98	25-1152-82	ThermoFisher Scientific	1:200	45 min, 4°C; 20 min for blood	https://www.thermofisher.com/antibody/product/CD115-c-fms-Antibody-clone-AFS98-Monoclonal/25-1152-82
	CD11c	N418	25-0114-82	ThermoFisher Scientific	1:300	45 min, 4°C; 20 min for blood	https://www.thermofisher.com/antibody/product/CD11c-Antibody-clone-N418-Monoclonal/25-0114-82
	F4/80	BM8	123114	BioLegend	1:200	45 min, 4°C; 20 min for blood	https://www.biolegend.com/en-us/products/pe-cyanine7-anti-mouse-f4-80-antibody-4070
	CD64 (FcγRI)	X54-5/7.1	139311	BioLegend	1:200	45 min, 4°C; 20 min for blood	https://www.biolegend.com/en-us/products/brilliant-violet-711-anti-mouse-cd64-fcgammari-antibody-9920
	CD3e	eBio500A2	48-0033-82	ThermoFisher Scientific	1:300	45 min, 4°C; 20 min for blood	https://www.thermofisher.com/antibody/product/CD3e-Antibody-clone-eBio500A2-500A2-Monoclonal/48-0033-82
	CD19	eBio1D3	48-0193-82	ThermoFisher Scientific	1:200	45 min, 4°C; 20 min for blood	https://www.thermofisher.com/antibody/product/CD19-Antibody-clone-eBio1D3-1D3-Monoclonal/48-0193-82
	MHC class II (I-A/I-E)	M5/114.15.2	14-5321-82	ThermoFisher Scientific	1:300	45 min, 4°C; 20 min for blood	https://www.thermofisher.com/antibody/product/MHC-Class-II-I-A-I-E-Antibody-clone-M5-114-15-2-Monoclonal/14-5321-82
	CD206 (MMR)	C068C2	141708	BioLegend	1:200	45 min, 4°C; 20 min for blood	https://www.biolegend.com/de-de/clone-search/apc-anti-mouse-cd206-mmr-antibody-7425?GroupID=BLG9506

Supplementary Table 1 | Inventory of all antibodies used throughout the study.
