# Supplementary material for: Distinct origins and niches determine the cellular responsiveness of CNS macrophages after repopulation
Source: Nat Immunol. 2026 Mar 18;27(5):961–74. doi: 10.1038/s41590-026-02457-y (PMC13132723; doi:10.1038/s41590-026-02457-y)
Supplement: Supplementary file 2 — Reporting Summary [file 41590_2026_2457_MOESM2_ESM.pdf]

nature portfolio

Corresponding author(s): Marco Prinz

Last updated by author(s): Feb 6, 2026

Reporting Summary

Nature Portfolio wishes to improve the reproducibility of the work that we publish. This form provides structure for consistency and transparency in reporting. For further information on Nature Portfolio policies, see our Editorial Policies and the Editorial Policy Checklist.

Statistics

For all statistical analyses, confirm that the following items are present in the figure legend, table legend, main text, or Methods section.

n/a	Confirmed	
☐	☒	The exact sample size (n) for each experimental group/condition, given as a discrete number and unit of measurement
☐	☒	A statement on whether measurements were taken from distinct samples or whether the same sample was measured repeatedly
☐	☒	The statistical test(s) used AND whether they are one- or two-sided<br>*Only common tests should be described solely by name; describe more complex techniques in the Methods section.*
☐	☒	A description of all covariates tested
☐	☒	A description of any assumptions or corrections, such as tests of normality and adjustment for multiple comparisons
☐	☒	A full description of the statistical parameters including central tendency (e.g. means) or other basic estimates (e.g. regression coefficient) AND variation (e.g. standard deviation) or associated estimates of uncertainty (e.g. confidence intervals)
☐	☒	For null hypothesis testing, the test statistic (e.g. F, t, r) with confidence intervals, effect sizes, degrees of freedom and P value noted<br>*Give P values as exact values whenever suitable.*
☒	☐	For Bayesian analysis, information on the choice of priors and Markov chain Monte Carlo settings
☐	☒	For hierarchical and complex designs, identification of the appropriate level for tests and full reporting of outcomes
☒	☐	Estimates of effect sizes (e.g. Cohen's d, Pearson's r), indicating how they were calculated

Our web collection on statistics for biologists contains articles on many of the points above.

Software and code

Policy information about availability of computer code

Data collection

Microscopy:
LAS X software v3.5.7.23225 was used for imaging with the Leica TCS SP8 X and Leica Thunder Imager.
BZ-X8000 Analyzer software was used for the Keyence BZ-X810 inverted fluorescence microscope.

FACS/Flow cytometry:
FACSDiva software (Becton Dickinson) was used for the sorting and data acquisition on the BD LSR Fortessa or BD Aria III.

RNA-Sequencing:
in bulk:
Library preparation with Illumina Nextera XT Sample Preparation. The sequencing run was performed on a HiSeq 1000 instrument (Control Software v2.2.68) using the indexed, 50 cycles single-read (SR) protocol and the TruSeq SBS v3 Reagents according to the Illumina HiSeq 1000 System User Guide. Image analysis and base calling were done by the Real Time Analysis Software (RTA) v3.7.17 software, which resulted in .bcl files, which were converted into fastq files with the bcl2fastq v2.20 software.
single cell:
Libraries were sequenced on a NextSeq1000 Sequencer (Illumina) appropriate for obtaining 20,000 reads per cell. The resulting fastq files were further processed using the Cell Ranger v7.1.0 pipeline (10x Genomics) for demultiplexing, read alignment to the mouse genome (GRCm39) and gene count determination.
scATAC-Sequencing:
Library preparation were performed according to the manufacturer’s protocol (Chromium Next GEM Single Cell ATAC Reagent kits v2, CG0004968, Rev B)
Raw ATAC-seq reads were processed using Cell Ranger ATAC (v2.2.0)

MRI and molecular mRI:
MRI imaging was performed on a Pharmascan 7T machine using Paravision 6.0.1 software.

Behavior:
Grip Strength test was performed using a Grip stremgth device by BIOSEB, France.
The corridor test is a custom designed test featuring blacck PVC walls, measuring 150 cm in length, 6 cm in width, and with walls standing 16 cm high. Along both the left and right walls, four fixed objects (3D-printed, custom-made; 2 cm high, 2 cm wide, 1 cm deep) were positioned 1 cm above the floor, spaced 18.5 cm apart. The objects' shape and color varied each test day.

Data analysis

Bulk RNA-seq data was aligned with STAR v2.7.11a to the mouse reference genome Gencode M36, mm10 or M39, depending on the date of the experiment. Counts were obtained using FeatureCounts v2.0.8. Data was analyzed using DESeq2 1.46.0.

scRNA-seq data was analyzed using RStudio (build 764, v2026.01, 2025.09 or 2025.05).
RStudio 4.4.0 2024.04.2+764 "Chocolate Cosmos" Release (e4392fc9ddc21961fd1d0efd47484b43f07a4177, 2024-06-05)
R packages: Seurat v.5.0.3, scDblFinder 1.16.060, clusterProfiler 4.10.0, EnhancedVolcano 1.20.0, BiomaRt 2.58.2, ggplot2 3.5.0

scATAC-seq data was analyzed using RStudio (build 764) following the Signac v1.16 vignette.

Microscopy: Leica LAS X software v3.5.7.23225
Image preparation:
Adobe Photoshop CS6 for clipping and thresholding
Adobe Illustrator 2025 for annotation and formatting
Manual quantification of histology was performed with Image J Fiji version 1.54p.
Semi-automatic quantification of histology was performed using QuPath 0.4.4.

Flow cytometry: Post-acquisition analysis was performed using FlowJo software, version 10.10.

Statistical analysis:
GraphPad Prism v10 was used for graph design and statistical analysis of histological, grip strength and flow cytometry data.
R studio was deployed to perform statistical testing for regression curves of repopulation kinetics, corridor test and correlation of grip strength with P-Sel signal (lawstats v3.6, gstats v2.1-4).

For manuscripts utilizing custom algorithms or software that are central to the research but not yet described in published literature, software must be made available to editors and reviewers. We strongly encourage code deposition in a community repository (e.g. GitHub). See the Nature Portfolio guidelines for submitting code & software for further information.

Data

Policy information about availability of data

All manuscripts must include a data availability statement. This statement should provide the following information, where applicable:

- Accession codes, unique identifiers, or web links for publicly available datasets
- A description of any restrictions on data availability
- For clinical datasets or third party data, please ensure that the statement adheres to our policy

All raw data are available in supplementary data files. High-throughput sequencing data is available through GEO (GSE318650, GSE295335, GSE294773, GSE318386, GSE318071, GSE318159, GSE294912, GSE295334). Accession codes are provided under Data availability.

Research involving human participants, their data, or biological material

Policy information about studies with human participants or human data. See also policy information about sex, gender (identity/presentation), and sexual orientation and race, ethnicity and racism.

Reporting on sex and gender	-
Reporting on race, ethnicity, or other socially relevant groupings	-
Population characteristics	-
Recruitment	-
Ethics oversight	-

Note that full information on the approval of the study protocol must also be provided in the manuscript.

Field-specific reporting

Please select the one below that is the best fit for your research. If you are not sure, read the appropriate sections before making your selection.

☒ Life sciences ☐ Behavioural & social sciences ☐ Ecological, evolutionary & environmental sciences

For a reference copy of the document with all sections, see nature.com/documents/nr-reporting-summary-flat.pdf

Life sciences study design

All studies must disclose on these points even when the disclosure is negative.

Sample size	No statistical methods were used to predetermine sample sizes. We ensured they were similar to those generally employed in the field (Masuda et al. 2022, Amann et al. 2024, Levard et al. 2024, Drieu et al. 2022).
Data exclusions	For all mouse experiments including depletion of macrophages, a depletion control at 24h after the last treatment was included, ensuring depletion of macrophages was successful (>85% for microglial density).<br><br>scRNAseq: detetcted doublets, low-quality cells, and cells expressing multiple cell-type specific marker genes were excluded (see methods) bulkRNAseq: genes with very low counts, immediate early genes, ribosomal and mitochondrial genes and pseudogenes were excluded (for details, see methods), additionally samples with significantly lower total aligned reads but same sequencing depth were excluded for poor quality (all included samples had >10 Million aligned reads per library, excluded samples only produced between 1-3 Million reads).<br><br>Stroke: blood flow downstream of the MCA was measured by laser doppler. Mice with an infarct volume below $5mm^3$, as determined by MRI, were excluded from further analysis (2/34).<br>Sequencing of stroke bulk data from sorted CAMs, produced two files (A3_ipsi and C2_ipsi) that had alignment < 10%, these samples were excluded from further analysis.
Replication	All experiments include greater than or equal to 3 biological replicates per group. Experiments, besides some high-througghput sequencing experiments to avoid batch effects, were split up into several groups and results were consistently replicable across groups.
Randomization	For all experiments, mice were randomly allocated to experimental groups, but with males and females being distributed evenly.
Blinding	All quantification experiments were performed in a blinded manner by assignment of unidentifiable numbers to mice, tissues and images for data acquisition and processing. Data labels and groups were only reinstated for statistical analysis. Quantification and imaging was not repeated following statistical analysis. Surgeries and outcome assessments were done blinded to treatment conditions.

Reporting for specific materials, systems and methods

We require information from authors about some types of materials, experimental systems and methods used in many studies. Here, indicate whether each material, system or method listed is relevant to your study. If you are not sure if a list item applies to your research, read the appropriate section before selecting a response.

Materials & experimental systems

n/a	Involved in the study
☐	☒ Antibodies
☒	☐ Eukaryotic cell lines
☒	☐ Palaeontology and archaeology
☐	☒ Animals and other organisms
☒	☐ Clinical data
☒	☐ Dual use research of concern
☒	☐ Plants

Methods

n/a	Involved in the study
☒	☐ ChIP-seq
☐	☒ Flow cytometry
☐	☒ MRI-based neuroimaging

Antibodies

Antibodies used

Target / Antigen Clone Catalog # Supplier / Source Dilution
VCAM-1 (CD106) M/K-2.7 BE0027 BioXCell 2 µg / µl
ICAM-1 (CD54) YN1/1.7.4 BE0020-1 BioXCell 2 µg / µl
VLA-4 (CD49d) PS/2 BE0071 BioXCell 2 µg / µl
HRP HRPN BE0088 BioXCell 200 µg in 100 µl i.p., every other day ×7 injections (matched to blocking Abs)
KLH LTF-2 BE0090 BioXCell 200 µg in 100 µl i.p., every other day ×7 injections (matched to blocking Abs)
P-selectin (for MPIO conjugation) polyclonal AF737 R&D Systems N/A
VCAM-1 (for MPIO conjugation) clone 429 553330 BD Biosciences N/A
Laminin polyclonal L9393 Merck Millipore 1:1000
Collagen IV polyclonal AB769 Millipore 1:500
IBA1 Gp311H9 234 308 Synaptic Systems 1:500

CD206 MR5D3 MCA2235 Biorad 1:500
CD31 polyclonal AF3628 R&D Systems 1:200
ICAM-1 (CD54) G-5 sc-8439 Santa Cruz 1:200
P-selectin (CD62P) polyclonal AF737 R&D Systems 1:200
GFP polyclonal ab13970 Abcam 1:1000
Ki-67 polyclonal ab15580 Abcam 1:250
MHC II M5/114.15.2 107622 BioLegend 1:200
mouse IgG polyclonal A10037 Thermo Fisher 1:500
rabbit IgG polyclonal ab175651 abcam 1:500
guinea pig IgG polyclonal A-11073 Molecular Probes 1:500
rat IgG polyclonal SA5-10029 Thermo Fischer 1:500
goat IgG polyclonal A11057 ThermoFisher 1:500
IBA-1 EPR16588 178846 Abcam 1:1000
CD3 CD3-12 MCA1477 Biorad 1:100
B220 (CD45R) RA3-6B2 557390 BD Biosciences 1:200
rabbit IgG polyclonal 4058-08 SouthernBiotech 1:300
Fc Block (CD16/32) 2.4G2 553141 BD Biosciences 1:25
CD11b (Integrin αM) M1/70 101237 BioLegend 1:300
CD45 30-F11 561037 BD Biosciences 1:200
Ly6C AL-21 561237 BD Biosciences 1:200
Ly6G 1A8 565964 BD Biosciences 1:300
Gr-1 (Ly6G/Ly6C) RB6-8C5 563849 BD Biosciences 1:300
CD115 (CSF1R / M-CSFR) AFS98 25-1152-82 ThermoFisher Scientific (eBioscience) 1:200
CD11c N418 25-0114-82 ThermoFisher Scientific 1:300
F4/80 BM8 123114 BioLegend 1:200
CD64 (FcγRI) X54-5/7.1 139311 BioLegend 1:200
CD3e eBio500A2 48-0033-82 ThermoFisher Scientific 1:300
CD19 eBio1D3 48-0193-82 ThermoFisher Scientific 1:200
MHC class II (I-A/I-E) M5/114.15.2 14-5321-82 ThermoFisher Scientific 1:300
CD206 (MMR) C068C2 141708 BioLegend 1:200

Validation

All primary anti-mouse antibodies used in the flow cytometry, western blot, mMRI or immunostainings have been validated for this application by the supplier and have been used in previous studies. Positive control stainings are shown wherever necessary. Please see supplementary files for full information on antibodies and antibody validation.

Animals and other research organisms

Policy information about studies involving animals; ARRIVE guidelines recommended for reporting animal research, and Sex and Gender in Research

Laboratory animals

C57BL/6JCrl (Charles River Laboratories)
B6.129S4-Ccr2tm1Ifc/J, JAX stock #004999

Cx3cr1CreERT2 (Yona et al. 2012)
Cxcr4CreERT2 (Werner et al. 2020)
HexbCreERT2 (Masuda et al. 2020, self-generated)
Mrc1CreERT2 (Masuda et al. 2022, self-generated)
Ccr2CreERT2 (Jax Stock #035229)
were crossed with R26tdT (JAX stock #007914) or R26YFP (Jax Stock #006148)

Wild animals

None.

Reporting on sex

Mice from both sexes were used in the present study and mice with different sex were split evenly across groups.

Field-collected samples

N.A.

Ethics oversight

Animal studies were approved by the Regional Councils of Freiburg, Germany and the French Ministry of Higher Education and Research and performed in accordance with the respective national, federal and institutional regulations.

Note that full information on the approval of the study protocol must also be provided in the manuscript.

Plants

Seed stocks: -

Novel plant genotypes: -

Authentication: -

Flow Cytometry

Plots

Confirm that:

☒ The axis labels state the marker and fluorochrome used (e.g. CD4-FITC).

☒ The axis scales are clearly visible. Include numbers along axes only for bottom left plot of group (a 'group' is an analysis of identical markers).

☒ All plots are contour plots with outliers or pseudocolor plots.

☒ A numerical value for number of cells or percentage (with statistics) is provided.

Methodology

Sample preparation	For the analysis of blood cells, around 100 µl of blood were collected from the right atrium and put in PBS containing 0,5 mol/L EDTA and afterwards lysed 3 times for 5 min with eBioscienceTM Red Blood Cell Lysis Buffer (ThermoFisher Scientific) before the staining procedure. All animals were perfused transcardially with ice cold PBS after blood collection and before isolating cells for flow cytometry. MG and CAMs were isolated from whole mouse brain with the notable exception of the experiments (from Fig. 3 onwards) where the choroid plexuses were removed through dissection under a stereomicroscope before continuing with digestion. For digestion, brains were homogenized and collected in HBSS containing 0.1 mg/ml DNAse I (Roche) and 0.2 mg/ml Liberase (Roche) and digested in a shaker at 37° C and 1000 rpm for 15 min. Afterwards, the brains were filtered through a 70µm filter. After spinning down for 5 min at 300 g, the cell pellets were collected in 37 % Percoll® solution (Sigma-Aldrich) and centrifuged for 30 min at 800g (no brakes) to remove the myelin. After myelin aspiration and removal of supernatant, the cell pellets were then collected in PBS containing 2 % FCS and 10 mM EDTA and used for the staining procedure.
Instrument	Cells were sorted using a BD Aria III or analyzed using a BD LSRFortessa (Becton Dickinson).
Software	Data were acquired with FACSDiva software (Becton Dickinson). Post-acquisition analysis was performed using FlowJo software, version 10.10
Cell population abundance	The cell population abundances are provided in the plots depicting the representative gating strategies.
Gating strategy	In all experiments, small debris was removed with the preliminary FSC/SSC gate. Single, living cells were obtained by doublet exclusion followed by the exclusion of dead cells using DAPI. Representative gating strategies are provided in the respective (supplementary) figures.

☒ Tick this box to confirm that a figure exemplifying the gating strategy is provided in the Supplementary Information.

Magnetic resonance imaging

Experimental design

Design type	resting state, 24 h post stroke, 5d post BLZ945 treatment
Design specifications	-
Behavioral performance measures	-

Acquisition

Imaging type(s): Structural

Field strength: 7 Tesla

Sequence & imaging parameters: T2-weighted images were acquired using a multislice multiecho sequence: TE/TR 33 ms/2500 ms. T2*-weighted sequences were used to control if animals underwent hemorrhages events. Two-dimensional time-of-flight angiographies (TE/TR 12 ms/7 ms) were acquired and analyses of the MCA angiogram were also performed to control the recanalization status of the MCA. Three-dimensional T2*- weighted gradient echo imaging with flow compensation (spatial resolution of 70 mm x 70 mm x 70 mm interpolated to an isotropic resolution of 70 mm), TE/TR 13.2ms/200 ms and a flip angle of 21° was performed to visualize MPIO.

Area of acquisition: Whole brain scan

Diffusion MRI: ☐ Used ☒ Not used

Preprocessing

Preprocessing software: Paravision 360.2.0.pl.1

Normalization: Each mouse served as its own control for signal since a baseline measuremnet was performed prior to antibody and prior to stroke application.

Normalization template: -

Noise and artifact removal: No noise or artifact removal

Volume censoring: no volume censoring

Statistical modeling & inference

Model type and settings: -

Effect(s) tested: -

Specify type of analysis: ☒ Whole brain ☐ ROI-based ☐ Both

Statistic type for inference (See Eklund et al. 2016): -

Correction: -

Models & analysis

n/a	Involved in the study
☒	☐ Functional and/or effective connectivity
☒	☐ Graph analysis
☒	☐ Multivariate modeling or predictive analysis
